# Understanding covid-19 outcomes among people with intellectual disabilities in England

**DOI:** 10.1186/s12889-023-16993-x

**Published:** 2023-10-25

**Authors:** Filip Sosenko, Daniel Mackay, Jill P. Pell, Chris Hatton, Bhautesh D. Jani, Deborah Cairns, Laura Ward, Angela Henderson, Michael Fleming, Dewy Nijhof, Craig Melville, CVD-COVID-UK/COVID-IMPACT Consortium

**Affiliations:** 1 The University of Glasgow, Glasgow, UK; 2 Manchester Metropolitan University, Manchester, UK; 3 The University of Dundee, Dundee, UK

**Keywords:** Covid-19, Intellectual Disabilities, Learning disabilities

## Abstract

**Background:**

Evidence from the UK from the early stages of the covid-19 pandemic showed that people with Intellectual Disabilities (ID) had higher rates of covid-19 mortality than people without ID. However, estimates of the magnitude of risk vary widely; different studies used different time periods; and only early stages of the pandemic have been analysed. Existing analyses of risk factors have also been limited. The objective of this study was to investigate covid-19 mortality rates, hospitalisation rates, and risk factors in people with ID in England up to the end of 2021.

**Methods:**

Retrospective cohort study of all people with a laboratory-confirmed SARS-CoV-2 infection or death involving covid-19. Datasets covering primary care, secondary care, covid-19 tests and vaccinations, prescriptions, and deaths were linked at individual level.

**Results:**

Covid-19 carries a disproportionately higher risk of death for people with ID, above their already higher risk of dying from other causes, in comparison to those without ID. Around 2,000 people with ID had a death involving covid-19 in England up to the end of 2021; approximately 1 in 180. The covid-19 standardized mortality ratio was 5.6 [95% CI 5.4, 5.9]. People with ID were also more likely to be hospitalised for covid-19 than people without ID. The main determinants of severe covid-19 outcomes (deaths and/or hospitalisations) in both populations were age, multimorbidity and vaccination status. The key factor responsible for the higher risk of severe covid-19 in the ID population was a much higher prevalence of multimorbidity in this population. AstraZeneca vaccine was slightly less effective in preventing severe covid-19 outcomes among people with ID than among people without ID.

**Conclusions:**

People with ID should be considered a priority group in future pandemics, such as shielding and vaccinations.

**Supplementary Information:**

The online version contains supplementary material available at 10.1186/s12889-023-16993-x.

## Introduction

Evidence from the UK from the early stages of the covid-19 pandemic showed that people with Intellectual Disabilities (ID) had higher rates of covid-19 mortality than people without ID. Public Health England estimated the risk ratio in the spring of 2020 to be 2.3–3.1, with an age-adjusted risk ratio of 4.1–6.3 [1]. The Office for National Statistics (ONS) estimated the age-adjusted mortality hazard ratio until November 2020 to be 3.7 and multiply-adjusted mortality hazard ratio to be 1.7 [2]. Williamson and colleagues found the multiply-adjusted mortality hazard ratio until August 2020 to be much higher at 8.2, with a similar figure for September 2020 – January 2021 [3]. As part of the QCOVID research, Clift and colleagues [4] estimated the multiply-adjusted mortality hazard ratio for people with ID (but not Down’s syndrome) in Spring 2020 to be 1.36, and for people with Down’s syndrome 9.80^1^. In Scotland, the crude risk ratio until August 2020 was 2.3 and multiply-adjusted risk ratio was 3.2 [5].

While there is agreement across these studies on a higher risk among people with ID, their estimates of the magnitude of risk vary widely; different studies used different time periods; and only early stages of the pandemic have been analysed. Populations under study also varied considerably in character and size: people with ID whose covid-19 related deaths were reported by hospitals, health professionals, family or carers [1], 6 million adults registered with a General Practice (GP) [4], 14 million adults registered with a GP [3], 30 million adults aged 30–100 alive at the beginning of 2020 who could be linked to Census 2011 in England [2], adults with ID in Scottish Census 2011 and a 5% random sample of adults without ID [5].

Furthermore, there is very little consistency in how ID was classified across these different analyses. For example, the Williamson study looked at people on the ID register in GP data. However, it is estimated that most people with ID are not on this register [6], resulting in under-coverage. In contrast, the ONS analysis and the QCOVID research included a large number of codes for specific conditions and syndromes considered to be strongly associated with intellectual disabilities, arguably resulting in over-coverage. Finally, the Scottish study used self- (or proxy-) reported ID to identify individuals with ID.

Additionally, different studies reported different mortality measures, making it difficult to compare findings: crude risk ratio [1, 5]; risk ratio adjusted for age [1]; risk ratio adjusted for age, sex and area deprivation [5]; hazard ratio adjusted for age [2]; hazard ratio adjusted for age, sex, residence type, geography, socio-economic and demographic factors, and health variables [2]; and hazard ratio adjusted for age, sex, ethnicity, and geographical location [3]. Estimates produced with different adjustments cannot be compared, while hazard ratio cannot be compared to a risk ratio (as the latter is derived from two cumulative incidences rather than two incidence rates).

Last but not least, the modelling of risk factors in the Williamson et al. [3] and ONS [2] studies included people who did not have SARS-CoV-2 infection at all as, unlike the present study, they did not have data about laboratory tests. Only under very specific and rather unrealistic assumptions would it not have biased the results. Furthermore, in both studies, time to event was ascertained from a fixed time-point rather than date of infection, which would introduce bias if the outcomes of interest varied by covid-19 variant (and other time-varying confounders) over the course of the pandemic.

The aim of the current study was to address the limitations of existing studies by accurately ascertaining cases over the main pandemic period. Importantly, the current study also aimed to deepen our understanding of risk factors. Pre-existing long-term conditions (LTCs) have been shown to have strong associations with severe covid-19 in general population studies [7], and some studies have examined associations between specific LTCs and covid-19 outcomes among people with ID [2, 3]. Similarly, polypharmacy has been found to be a risk factor for severe covid-19 outcome in the general population [8, 9]. However, no studies have examined the effects of multimorbidity and polypharmacy on severe covid-19 among people with ID, who are known to have higher rates of both [10, 11]. Furthermore, no studies have examined the associations between vaccination status, vaccine type, and severe covid-19 in people with ID.

The current paper aims to address the gaps and limitations in research outlined above. Specifically, the research questions were:

- How does covid-19 mortality and hospitalisations among people with ID compare with that of people without ID?
- Are there significant differences in the associations between clinical risk factors and risk of hospitalisation and death following SARS-CoV-2 infection in people with and without ID?
- Which factors are key drivers of severe covid-19, in both the ID and non-ID populations?
- What factors are responsible for the higher probability of severe covid-19 among people with ID?

## Methods

### Study design

A population-based retrospective cohort study was undertaken using routinely collected electronic health record (EHR) data. It was conducted on behalf of the CVD-COVID-UK/COVID-IMPACT Consortium (coordinated by the BHF Data Science Centre). Approvals were obtained from the research consortium and from the University of Glasgow Ethics Committee.

The study population was all people in England who had a confirmed SARS-CoV-2 infection. SARS-CoV-2 infection was defined as a positive laboratory test, or a death involving covid-19^2^. Since the vast majority of recorded laboratory tests were of the polymerase chain reaction (PCR) type [13], we refer to them as ‘PCR tests’ in this paper. Subjects were classified as having ID or not based on SNOMED CT codes recorded in primary care data. (As such, the correct term for this population is ‘people identified in their GP records as having ID’ rather than ‘people with ID’. We use the latter term for brevity, however). The list had 378 codes and was a merger of 360 codes in GDPPR forming the ‘Learning Disability’ cluster ([14], cluster LD_COD version 2021/12/21) and 259 codes recommended by NHS England for identification of people with ID [15]. The number of people with ID identified using this list of codes was 29% higher than the number of people on ID register and constituted 0.59% of all people in the dataset (cf. 0.46% on ID register).

This analysis was performed according to a pre-specified analysis plan published on GitHub, along with the phenotyping and analysis code (https://github.com/BHFDSC/CCU030_01). The list of codes used for identifying people with ID is in Table A7 in Additional file 1.

### Data

Anonymised individual-level data were accessed through the Secure Data Environment (SDE), provided by NHS England in England and accessed via the BHF Data Science Centre [16]. Datasets that were linked for the current project included primary care (GDPPR: General Practice Extraction Service Data for Pandemic Planning and Research), secondary care (HES: Hospital Episode Statistics, CHESS: COVID 19 Hospitalisation in England Surveillance System), covid-19 laboratory tests (SGSS: Second Generation Surveillance System) and vaccination status (Covid 19 vaccination events), deaths (Civil Registry Deaths), and prescribing/dispensing (NHS BSA Dispensed Medicines). The linkage was conducted using an anonymized version of the NHS number. The merged dataset of unique individuals with a laboratory-confirmed SARS-CoV-2 infection (or covid-19 death) by the end of 2021 had 10,660,640 records, including 56,870 people with ID. The dataset contained the earliest confirmed infection and its outcome within 28 days (recovery without hospitalisation; hospitalisation without death; hospitalisation with death; death without hospitalisation).

It needs to be highlighted that the primary care dataset available for this study (GDPPR) was a bespoke extract and as such it did not contain all information about health conditions diagnosed or treated in primary care [17]. Therefore, prevalences of long-term health conditions according to the data could be lower than the actual population prevalences.

### Data analyses

The outcomes were death involving covid-19 (defined as an ICD-10 code of U07.1 or U07.2 recorded in any position on the death certificate) and hospitalisation involving covid-19 (defined as admissions with ICD-10 diagnosis of U07.1 or U07.2, not restricted to primary diagnosis). The exposures of interest were ‘complex multimorbidity’ [18], polypharmacy, vaccination status and vaccine type. An indicator of complex multimorbidity (hereafter called just ‘multimorbidity’ for brevity) was constructed as at least three of 36 conditions (including 35 commonly experienced LTCs [19] and ID), of which at least one is physical [10], recorded in either primary or secondary care records within one year before the SARS-CoV-2 infection. The presence of polypharmacy was based on five or more unique prescription medications used [20] at any point in the time window from 255 days before the positive PCR test to 15 days before the test, as per study by McKeigue and colleagues [8]. Demographic covariates included age, sex, ethnicity, and socioeconomic deprivation (measured by the Index of Multiple Deprivation, IMD [21]).

In the analysis of risk, the pandemic was divided into two periods: one up to the end of 2020, when the vaccination programme was just starting, and another covering all of 2021. We decided to not include data from 2022 as that period was dominated by a much milder Omicron variant [22]. At the time of the analysis no data were available on the variant at the individual level nor the dominant variant at that stage of the pandemic, meaning that this aspect could not be controlled for in the modelling. We also excluded cases where the individual did not have any primary care record, as this meant that it was not possible to determine their ID status.

Data management and data analysis were conducted using Python v3.7, SQL, and R v4.03. Phenotypes for long-term conditions were sourced from the Cambridge Multimorbidity Score [23]. Crude and age-standardized mortality and hospitalisation risk ratios are reported. Age-specific rates were inspected for inconsistent relationships before standardization [24].

With regards to the modelling of associations between risk factors and severe covid-19 outcome, we decided that logistic regression provides a more appropriate modelling framework than survival analysis. The main reason was that the former allowed us to use a much larger number of records. Survival modelling of severe covid-19 outcomes would need to exclude people who died of covid-19 without a positive PCR test, as well as people who had their PCR test after being admitted to hospital – an overall loss of half of the sample, or 22,000 records in the case of people with ID. This would result not only in decreased precision of estimates and their standard errors, but it would likely entail a degree of bias in coefficients, as records that would need to be excluded from survival modelling had somewhat different characteristics from their included equivalents, i.e. people who were hospitalised or died at least 1 day after a positive PCR test. (The former tended to be older and have a higher mean count of LTCs).

A secondary rationale was that counting time at risk from a positive covid-19 test to hospitalisation / death would introduce a sizeable measurement error, since the real starting point is the moment of infection rather than the moment of positive test. In the best-case scenario this would only increase standard errors without biasing coefficients; in the worst-case scenario this would also bias coefficients. We have, however, conducted survival modelling as a sensitivity test of the logistic modelling. While hazard ratios and odds ratios are not directly comparable, they should be similar when the outcome of interest is rare [25], which was the case in our study.

The outcome in the logistic modelling was the presence or absence of severe covid-19 outcome while predictors included demographic (age, sex, ethnicity, area deprivation) and other (vaccination type, multimorbidity and polypharmacy) factors of relevance. The population was adults who had a confirmed SARS-CoV-2 infection or whose death involved covid-19 without a PCR test. We excluded children from modelling as our exploratory analysis established that the relationship between age and severe covid-19 is different among children than among adults. (Among children, the risk of severe covid-19 outcome decreases as age increases. Further research would be required to find out if this is an artefact of the PCR testing regime, or there is some kind of ‘healthy survivor effect’).

To explore the question of what factors are responsible for the difference in the risk of severe covid-19 between people with ID and without ID, and within that whether it is the different prevalence or the different effect of those factors that matter, we employed the Blinder-Oaxaca decomposition technique [26]. It compares a given outcome across two groups and allows for separating the effect of a different prevalence of a given factor on the outcome from the effect of that factor’s different coefficient on the outcome.

## Results

### Covid-19 mortality

Two thousand and forty people with ID had a death involving covid-19 up to the end of 2021. Covid-19 deaths constituted a somewhat higher proportion of all deaths among people with ID than in the non-ID population. In 2020, over a quarter (27.6%) of all deaths among people with ID involved covid-19, versus 18.9% among people without ID.

In terms of the crude probability of dying from covid-19, people with ID were over twice (2.1) as likely to have a death involving covid-19 than people without ID (Table 1). One in 180 people with ID – 0.56% of this population – had a death involving covid-19 until the end of 2021. Cumulative incidence was lower in 2021 than in 2020 in both populations, reflecting the roll-out of vaccinations from December 2020.

**Table 1:** Covid-19 mortality, by ID status and year (SDE database)

	2020		2021		Whole period	
	ID	No ID	ID	No ID	ID	No ID
Population size^a^	365155	61086510	365890	61675315	365895	61675400
Number of covid-19 deaths	1110	84890	930	75340	2040	160230
Cumulative incidence (%)	0.30	0.14	0.25	0.12	0.56	0.26
That is 1 in …	329	720	394	819	180	385
Crude Risk Ratio	2.2		2.1		2.1	
Number of expected covid-19 deaths	178	reference population	185	reference population	363	reference population
Covid-19 SMR [95% CI]	6.2 [5.9, 6.6]		5.0 [4.7, 5.4]		5.6 [5.4, 5.9]	

^a^Number of records in the dataset

The covid-19 standardized mortality ratio was 5.6 [95% CI: 5.4, 5.9] over the whole period, much higher than the crude risk ratio. This has been driven by much higher covid-19 mortality rates among younger people with ID than younger people without ID (Additional file 1: Table A3), combined with the higher proportion of younger people in the ID population than in the no-ID population (Additional file 1: Table A2).

### Covid-19 hospitalisations

People with ID were more likely to be hospitalised for covid-19 than people without ID: around 1 in 50 compared with 1 in 121, up to the end of 2021 (Table 2). In 2020 the crude risk ratio was 2.5 while the standardized incidence ratio was 4.3. Nominally, cumulative incidence of hospitalisation was similar in 2020 and 2021 in each of the groups but considering that covid-19 hospitalisations really started in March 2020, it can be said that incidence decreased somewhat in 2021.

**Table 2:** Covid-19 hospitalisations, by ID status and year (SDE database)

	2020		2021		Whole period	
	ID	No ID	ID	No ID	ID	No ID
Population size^a^	365155	61086510	365890	61675315	365895	61675400
Covid-19 hospitalised^b^	3590	236440	3745	272515	73340	508955
Cumulative incidence (%)	0.98	0.39	1.02	0.44	2.01	0.83
That is 1 in …	102	258	98	226	50	121
Crude Risk Ratio	2.5		2.3		2.4	
Number of expected covid-19 hospitalisations	838	reference population	1187	reference population	2024	reference population
Covid-19 SIR [95% CI]	4.3 [4.2, 4.4]		3.2 [3.1, 3.3]		3.6 [3.5, 3.7]	

^a^Number in the analysis dataset

^b^Unique individuals

Taking together covid-19 deaths and covid-19 hospitalisations (i.e. “severe covid-19 outcome”), the crude incidence risk ratio in 2020 was 2.5 while the standardized incidence ratio was 4.4 (Additional file 1: Table A5).

### Risk factors for severe covid-19 outcome

#### Characteristics of the population with a confirmed infection

Patterns regarding sex, area deprivation, multimorbidity and polypharmacy were similar among people with a confirmed SARS-CoV-2 infection and the whole population of England alive on 1 January 2020 (see Table 3, and Tables A1 and A2in Additional file 1). Those with ID were more likely to be male and more likely to live in a more deprived area than people without ID. The prevalence of polypharmacy and multimorbidity among adults with ID were over twice the level among adults without ID; the gap was even bigger among children. Among those whose death involved covid-19 however, the prevalence of multimorbidity and polypharmacy was almost identical between people with ID and without ID (Additional file 1: Table A3).

**Table 3:** Demographic and health characteristics of adults with confirmed SARS-CoV-2 infection (SDE database)

	**No ID**	**ID**	***p* value**
N in the analysis dataset	8408930	45820	
Age at covid-19 (median [IQR])	41.79 [29.95, 54.99]	40.79 [27.99, 57.81]	< 0.001
Female (%)	54.0	42.2	< 0.001
Ethnicity (%)			< 0.001
White	82.8	87.7	
Asian	9.2	6.5	
Black	3.7	3.1	
Mixed	1.9	1.6	
Other	2.4	1.0	
IMD decile (mean (SD))	5.36 (2.85)	4.59 (2.74)	< 0.001
Percent vaccinated at least once	50.8	43.3	< 0.001
Vaccine, if any: (%)			< 0.001
AstraZeneca	41.4	58.7	
Moderna	6.6	2.9	
Pfizer	51.9	38.4	
Percent affected by multimorbidity*	13.1	32.4	< 0.001
Count of Long-Term Conditions (mean (SD))**	1.32 (3.18)	2.60 (4.20)	< 0.001
Percent affected by polypharmacy***	20.9	55.6	< 0.001
Count of prescription medications (mean (SD))	2.70 (3.84)	6.28 (5.07)	< 0.001

*Note*: p-values were obtained using t-tests for continuous variables (ones with a mean and SD presented) and Chi-squared tests for all other variables

^*^Multimorbidity: 3 + LTCs (including ID) of which at least one is physical [10]

^**^The count did not include ID

^***^5 + prescription medications

Adults with ID were less likely to have been vaccinated than adults without ID while the opposite was true for children.

### Risk factors for severe covid-19 outcome

The modelling of risk factors showed (Table 4) that, net of the effect of other risk factors, the risk (odds) of severe covid-19 outcome (i.e. hospitalisation or death):

- is higher in older age groups in people with and without ID, but less so in the ID population. This does not mean that older people with ID are at smaller risk than their peers without ID; it is due to the fact that the crude risk among younger people with ID is already high, so there is a ‘ceiling’ effect with regards to age in the ID population
- is lower for women in both groups
- is higher for people from non-White ethnic backgrounds, in both groups
- is lower for the non-ID population living in less deprived areas, while there seems to be no relationship between area deprivation and severe covid in the ID population. (Lack of statistical significance is likely due to lack of effect rather than due to an insufficient number of records).
- is lower for people who have been vaccinated, in both groups
- varies by vaccine, with Moderna being the most effective, followed by Pfizer and AstraZeneca. AstraZeneca was somewhat less effective among people with ID than among people without ID
- is higher for people affected by multimorbidity (rises with the count of LTCs), in both groups. However, the effect of multimorbidity on the risk of severe covid-19 outcome varies by the extent of polypharmacy: the effect is stronger when polypharmacy score is low than when it is high
- is higher for people affected by polypharmacy (rises with the count of prescription medicines), in both groups. However, the effect of polypharmacy on the risk of severe covid-19 outcome varies by the extent of multimorbidity: the effect is stronger when multimorbidity score is low than when it is high.

**Table 4:** Results from a logistic model predicting severe covid-19 outcome among the confirmed infected adults, fit separately to ID and non-ID populations (SDE dataset)

	ID			No ID		
	Odds ratio	95% CI	*p*-value	Odds ratio	95% CI	*p*-value
Age at covid	1.03	1.03, 1.03	0.000	1.04	1.04, 1.04	0.000
Female	0.78	.73, .83	0.000	0.74	.74, .75	0.000
Ethnicity						
White	(base)			(base)		
Asian	1.92	1.69, 2.17	0.000	1.86	1.84, 1.89	0.000
Black	1.87	1.58, 2.23	0.000	2.25	2.21, 2.29	0.000
Mixed	1.13	.86, 1.49	0.390	1.69	1.65, 1.74	0.000
Other	1.84	1.36, 2.5	0.000	2.09	2.04, 2.14	0.000
IMD decile	^a^			0.97	.96, .97	0.000
Vaccination						
None	(base)			(base)		
AstraZeneca	0.38	.34, .41	0.000	0.27	.27, .27	0.000
Moderna	0.09	.04, .17	0.000	0.08	.08, .09	0.000
Pfizer	0.17	.15, .19	0.000	0.19	.19, .19	0.000
Count of long-term conditions^b^	1.61	1.59, 1.63	0.000	1.55	1.55, 1.55	0.000
Count of prescription medicines	1.08	1.07, 1.09	0.000	1.10	1.10, 1.10	0.000
Interaction of the above two counts	0.98	0.98, 0.98	0.000	0.98	0.98, 0.98	0.000
Constant	0.01	0.01, 0.01	0.000	0.00	0.00, 0.00	0.000
Observations	45540			8250210		

^a^Excluded from the ID model due to lack of statistical significance

^b^The count did not include ID

**Fig. 1:**
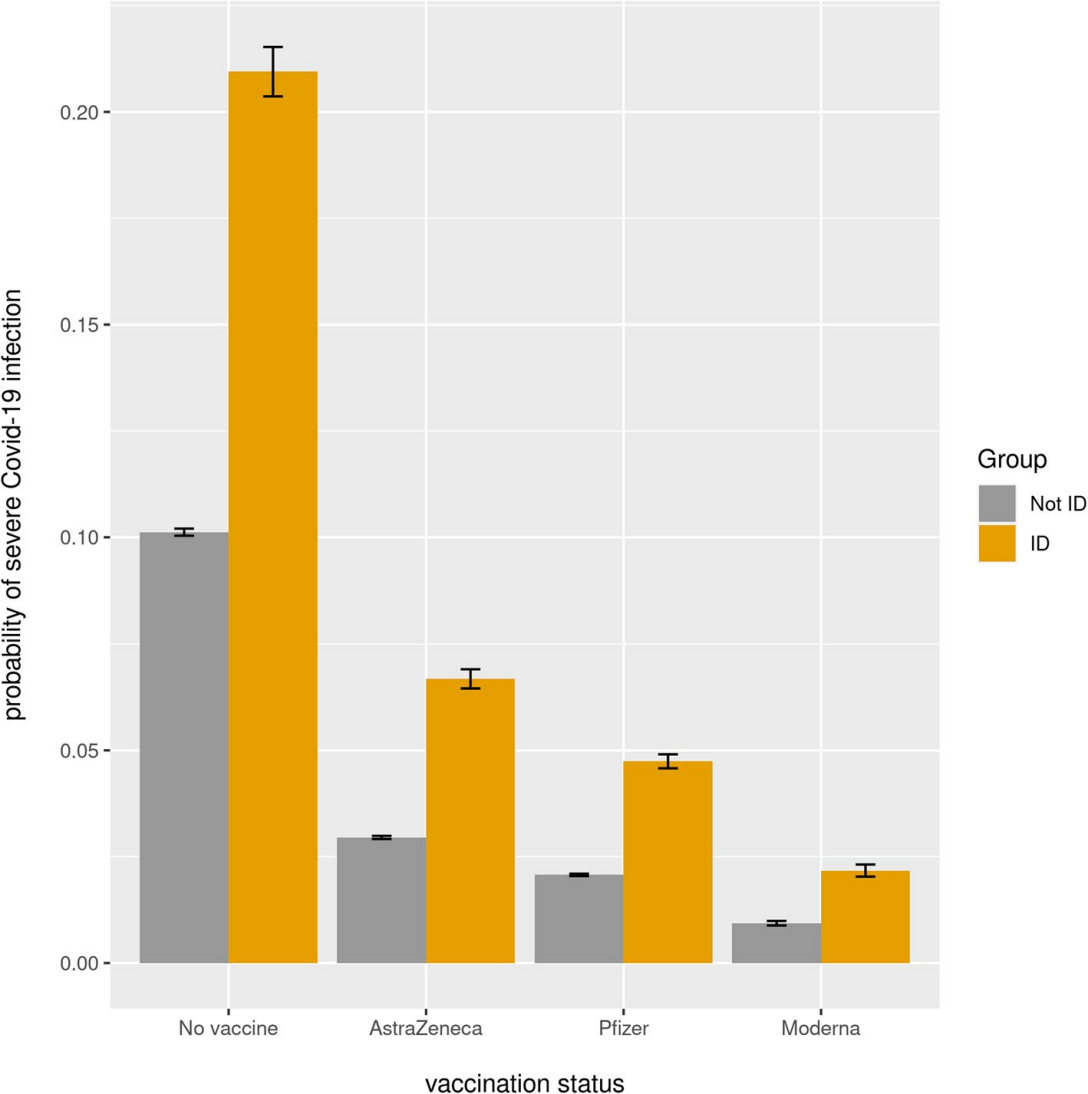
Predicted probability of severe covid-19 outcome in 75-year-old White men with a confirmed SARS-CoV-2 infection (SDE dataset)

To illustrate the effect of vaccination we calculated predicted probabilities of severe covid-19 outcome for a 75-year-old White man with a confirmed infection, a median number of health conditions, a median number of prescription medications, and living in an area of IMD decile 5. For this kind of individual, the risk decreases approximately threefold after AstraZeneca and tenfold after Moderna (Fig. 1). The magnitude of the effect is similar regardless of ID status.

Results of our sensitivity analysis—in the form of Cox regression—were similar to the logistic regression results (Table 5).

**Table 5:** Results from a Cox proportional hazards model of time elapsed from positive PCR test to outcome (hospitalisation, death if there was no hospitalisation, or recovery without hospitalisation) within 28 days, among adults (SDE dataset)

	ID			No ID		
	Hazard ratio	95% CI	*p*-value	Hazard ratio	95% CI	*p*-value
Age at covid	1.02	1.02, 1.02	0.000	1.03	1.03, 1.03	0.000
Female	0.82	.77, .87	0.000	0.70	.69, .70	0.000
Ethnicity						
White	(base)			(base)		
Asian	1.54	1.32, 1.81	0.000	1.80	1.78, 1.83	0.000
Black	1.66	1.48, 1.86	0.000	1.69	1.68, 1.71	0.000
Mixed	1.07	0.81, 1.40	0.643	1.48	1.44, 1.52	0.000
Other	1.73	1.32, 2.28	0.000	1.80	1.76, 1.84	0.000
IMD decile	0.99	0.98, 0.99	0.009	0.97	.97, .97	0.000
Vaccination						
None	(base)			(base)		
AstraZeneca	0.51	.47, .55	0.000	0.41	.41, .41	0.000
Moderna	0.09	.04, .23	0.000	0.11	.10, .12	0.000
Pfizer	0.26	.23, .30	0.000	0.29	.28, .29	0.000
Count of long-term conditions^a^	1.26	1.24, 1.27	0.000	1.25	1.25, 1.25	0.000
Count of prescription medicines	1.02	1.01, 1.03	0.000	1.03	1.03, 1.03	0.000
Interaction of the above two counts	0.99	0.99, 0.99	0.000	0.99	0.99, 0.99	0.000
Observations	22509			2960599		
Number of events	4816			340245		

^a^The count did not include ID

It was not possible to investigate the extent to which the probability of severe covid-19 was accounted for by the risk factors considered, due to the lack of R-squared in logistic modelling and survival regression. Royston’s R^2^D – a pseudo R-squared ranging from 0 to 1, commonly used in survival modelling—had the value of 0.46 in the ID model and 0.58 in the no-ID model.

### Which risk factors influence severe covid-19 outcomes the most

While the above-presented model shows that several factors were statistically significant predictors of increased risk of severe covid-19 (net of the effect of other factors in the model), and that a few factors had a somewhat different effect on risk among people with ID than among people without ID, the coefficients themselves do not tell us about the relative contribution of each factor to the outcome. To investigate this, we have conducted hierarchical logistic regression using the same predictors as in the model presented above and looked at three measures of model fit: MacFadyen’s Pseudo R-squared, Akaike Information Criterion and Bayesian Information Criterion. Table 6 below shows that the three factors driving the outcome the most in both ID and non-ID populations were age, multimorbidity and vaccination status, in this order. (Pseudo R-squared and AIC/BIC change in a major way when these factors are added to the model, unlike other factors). However, age was a less strong determinant in the ID population than in the non-ID population: pseudo-R increased less in the former when age was added to the model. Polypharmacy was a marginal driver once the effect of multimorbidity was accounted for.

**Table 6:** Pseudo R-squared and Information Criteria from hierarchical logistic regression predicting the risk of severe covid-19 (SDE dataset)

	**ID**			**No ID**		
	Pseudo R-squared	AIC	BIC	Pseudo R-squared	AIC	BIC
Intercept-only	0.000	40646.9	40655.7	0.000	3985720.5	3985734.4
As above + age	0.114	36003.2	36020.6	0.234	3052399	3052426.8
As above + sex	0.115	35975.4	36001.6	0.236	3044470.9	3044512.7
As above + ethnicity	0.119	35809.8	35870.9	0.243	3017739.1	3017836.6
As above + IMD decile	0.120	35771.1	35840.9	0.250	2989951	2990062.4
As above + vaccination data	0.165	33971.4	34084.9	0.298	2796673.4	2796854.5
As above + multimorbidity	0.252	30409	30531.2	0.366	2528679.3	2528874.3
As above + polypharmacy	0.255	30293.8	30424.7	0.370	2509799.9	2510008.8

However, it is worth pointing out that age and multimorbidity are also key drivers of non-covid-19 deaths (see Additional file 1: Table A6).

### Which risk factors are responsible for the higher probability of severe covid-19 among people with ID

**Fig. 2:**
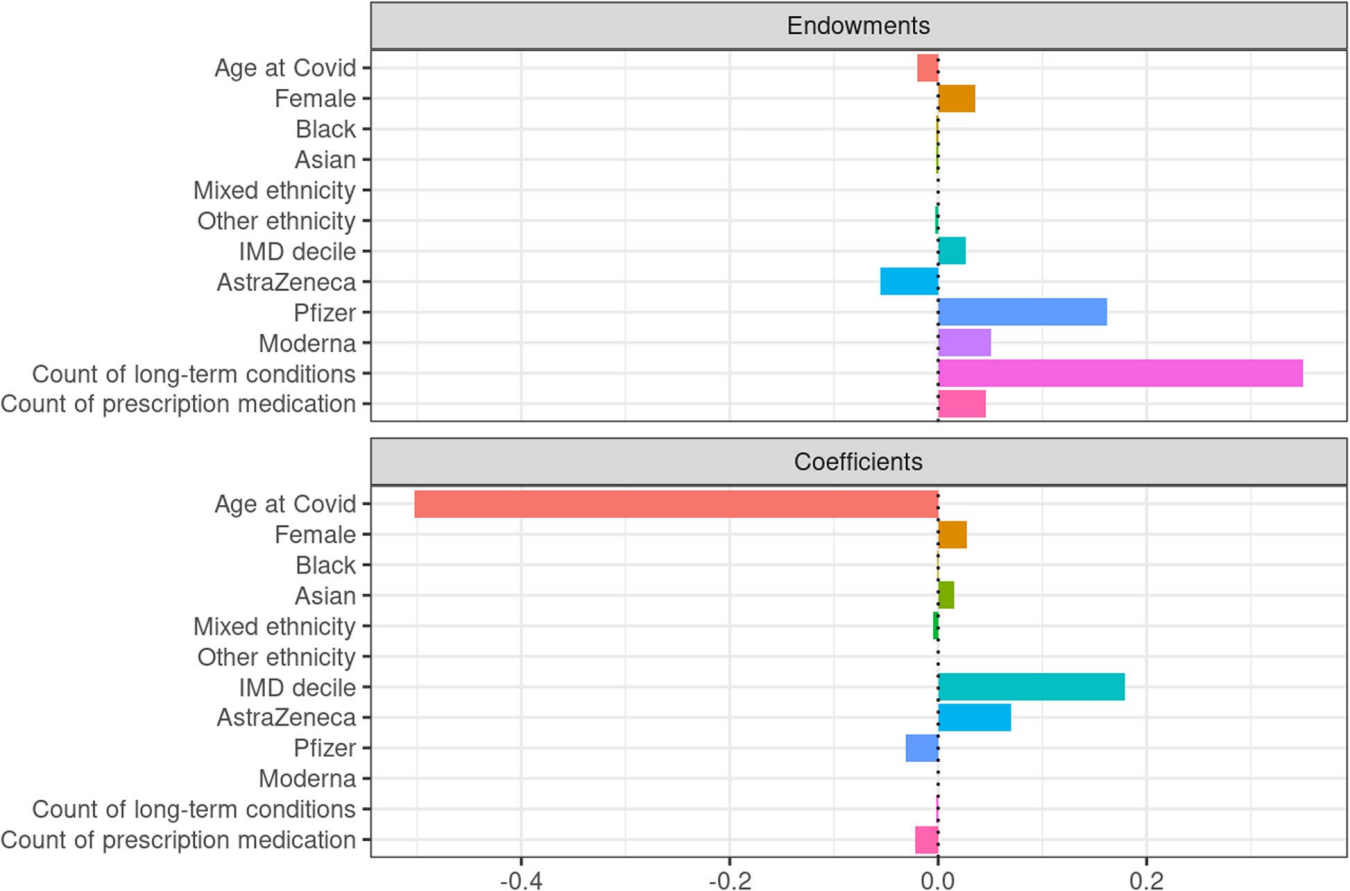
Results of Blinder-Oaxaca decomposition (SDE dataset). Note: count of long-term conditions did not include ID

The Blinder-Oaxaca decomposition indicated that around 40% of the difference in the risk of severe covid-19 between people with ID and without ID was explained by differences in prevalence while 60% was explained by differences in coefficients. More specifically, Fig. 2 shows that the higher risk of severe covid-19 among people with ID was driven mainly by a much higher prevalence of multimorbidity. Secondary factors included lower prevalence of Pfizer vaccination in the ID group, a weaker effect of AstraZeneca in the ID group, and different coefficients for area deprivation (living in a less deprived area was a protective factor among people without ID but not among people with ID). On the other hand, the gap in risk would have been considerably bigger had it not been for the fact that the effect of age on risk was weaker among people with ID.

(In the plot below the ‘Endowments’ part refers to the effect of prevalence. The bars to the right from the zero point on the horizontal axes indicate what contributes to the difference in risk while bars to the left from the zero point indicate what reduces the difference in risk. The scale on the horizontal axis refers to the difference in log odds of severe covid-19 outcome; The probability of severe covid-19 outcome among people with a confirmed SARS-CoV-2 infection was 0.164 in the ID group and 0.065 in the non-ID group, a difference in log odds was 1.04).

## Discussion

Below we discuss our findings and compare them with findings of previous relevant studies.

It is important to emphasise that it is difficult to know whether differences in findings are due to different time periods, different study designs, different sizes and characteristics of samples, or limitations of specific studies. However, we reiterate that our main aims were to obtain valid estimates for the whole pre-Omicron pandemic period and to deepen our understanding of risk factors, rather than to improve on estimates from the first 1–2 waves of the pandemic.

### Covid-19 mortality

The study estimates that 2,040 people with ID had a death involving covid-19 in England up to the end of 2021; higher than the estimate of 1,675 from the LeDeR programme [27]. This discrepancy is likely due to the fact that LeDeR relies on an incomplete source of data [1]. Our study found the crude mortality risk ratio in 2020 to be 2.2, a slightly lower figure than the 2.3–3.1 found in the PHE study in spring 2020 [1] and very similar to the crude risk ratio found in Scotland until August 2020 [5]. Overall, there is correspondence between these findings as a higher estimate of mortality (in PHE) would be expected to be found in the initial period of the pandemic, before treatments were introduced.

Our adjusted odds ratio of covid-19 death in 2020 was 3.2 (3.19 when converted to risk ratio [28]), identical to the Scottish adjusted mortality ratio in 2020. Our study also found the SMR to be around 5.6 over the whole period. In our view, however, the crude rate ratio provides a more valid comparison of mortality in the two populations than the SMR or other age-adjusted measures. Direct age-standardization is helpful when the differences in age structures between two populations result from factors not concerning the age-mortality relationship in each of them. For example, the population of one country may be on average younger than of another because it went through a war and a subsequent ‘baby boom’. Indirect age-standardization, similarly, is helpful when differences in age-specific mortality rates between two populations are due to external factors, such as the quality of health care, diet or prevalence of smoking. Age standardization (direct or indirect) is problematic in the ID population because its younger age profile, or higher mortality rates at younger ages, are not mainly due to such external factors but due to a different age-mortality relationship: more people with ID die at a younger age *because of their ID*. (Particularly due to comorbidities related to ID; not questioning the scope for further gains in life expectancy through better health care). Thus, *to the extent that shorter life expectancy among people with ID is due to internal rather than external factors*, age standardisation (direct or indirect) may inflate the effect.

Importantly, the crude covid-19 mortality risk ratio was higher in 2020 than the crude *non*-covid-19 mortality risk ratio (2.2 and 1.4 respectively); see Table A4 in Additional file 1. Similarly, the covid-19 SMR was higher (6.2) than non-covid-19 SMR (3.5). This means that covid-19 carries a disproportionately higher risk for people with ID, above the already higher risk of dying from other causes, in comparison to the no-ID population.

We found that risk of covid-19 death decreased in 2021, but more so in the ID population. This might be because mortality in this group in 2020 was potentially influenced by poorer access to appropriate treatment once hospitalised. One study using data from the first wave of the pandemic found that “despite having more severe symptoms on admission and similar rates of complications, patients with ID were less likely to be treated with non-invasive ventilation, tracheal intubation, or be admitted to an Intensive Care Unit setting” [29]. Another study found instances of inappropriate use of Do Not Attempt Cardiopulmonary Resuscitation (DNACPR) orders [30].

Since our measures of risk are based on cumulative incidence rather than time-to-event data, none of them can be compared to the hazard ratios reported by the ONS [2] and Williamson and colleagues [3].

### Covid-19 hospitalisations

The study found that around one in 50 people with ID had a hospitalisation involving covid-19 up to the end of 2021; a first estimate of this kind. The crude hospitalisation risk ratio was slightly higher than the crude mortality ratio (2.4 and 2.2 respectively). In contrast, the SIR (3.6) was considerably lower than SMR (5.6). As with mortality, the incidence decreased in 2021 relative to 2020.

### Covid*-*19 risk factors

The study has found that age, multimorbidity and vaccination status are key determinants of severe covid-19 in both ID and non-ID populations. Age and multimorbidity are also key determinants of non-covid-19 mortality. Being vaccinated had a similar protective effect in both populations, but notably the AstraZeneca vaccine was somewhat less effective in protecting people with ID than people without ID.

With regards to factors responsible for the risk of severe covid-19 being higher in the ID population, our analysis showed that the key factor was a much higher prevalence of multimorbidity in this population.

This is the first study to demonstrate a relationship between multimorbidity experienced by adults with ID and a health outcome, mortality. Previous research on multimorbidity and ID has focussed on describing the extent of multimorbidity by adults with ID [10, 31, 32] but there has been no previous evidence that multimorbidity is associated with negative health outcomes of adults with ID. The results of the decomposition analysis demonstrated that multimorbidity was the leading factor responsible for the increased risk of severe Covid-19 outcomes experienced by adults with ID, compared to adults without ID. This highlights the potential importance of multimorbidity as a target for health improvement strategies to reduce the inequalities in mortality experienced by adults with ID. However, future studies should examine the relationships between multimorbidity and non-covid outcomes. Although our findings highlight multimorbidity as a potential target for health improvement strategies, we should not assume that strategies developed from generic multimorbidity research will have an equitable impact on multimorbidity experienced by people with ID [33]. This is made clear in the results of our study because of the very early onset of multimorbidity for people with ID, compared to people without ID. In our data (for the beginning of 2020), the prevalence of complex multimorbidity was already 13% among children with ID (vs 2% among children without ID). Of people with ID who had complex multimorbidity, a quarter were aged 23 or under whereas a quarter of people without ID who had complex multimorbidity were aged 45 or under.

The earlier onset of multimorbidity in people with intellectual disabilities has been attributed to neurodevelopmental and health conditions associated with genetic and other causes of ID [34]. However, the multiple social disadvantage due to socio-economic deprivation [35], social isolation [36] and neighbourhood effects [37] experienced by people with ID are likely to be contributing to the early onset of multimorbidity. High rates of physical inactivity, sedentary behaviours and unhealthy dietary habits due to the lack of social support to allow people with ID to make positive lifestyle choices have also been shown to partly explain why people with ID experience such high rates of multimorbidity at a younger age [32].

We have described the different relationship between age and multimorbidity, the potential effects of multiple social disadvantages and the distinct pattern of causes underlying multimorbidity experienced by people with ID. Therefore, policy makers and research funders interested in health improvement should invest resources to inform the development of multimorbidity prevention and management strategies that are tailored to the needs of people with ID. More immediately, the combination of high levels of multimorbidity among people with ID with multimorbidity being a major risk factor for severe covid-19 suggests that people with ID should be automatically eligible for covid-19 booster vaccinations.

### Strengths and limitations of the study

The study had a strength in terms of coverage: it was able to include not only people who are on the ID register but also some people with confirmed ID who are not on this register. The number of people with ID in our study was 29% higher than the number on the register. While our ID prevalence of 0.59% is still considerably below the 2% estimated by PHE [6], it nevertheless represents an improvement in coverage over just using the ID register (0.46%). On the other hand, our ID population likely had fewer ‘false positives’ than the ONS and QCOVID research [2, 4], which used many conditions that do not necessarily indicate ID.

The study also covered the whole pre-Omicron time of the pandemic rather than just the first 1–2 waves. Additionally, our study benefited from a much larger number of observations in the database than any of the previous studies.

The strength of our modelling was that it was able to focus on those with a confirmed SARS-CoV-2 infection; earlier studies did not have testing information (e.g. [3]) and inevitably conflated those who were not infected at all with those who were infected but did not have a PCR test. Additionally, we were able to conduct a sensitivity analysis, increasing the robustness of findings.

One limitation of the study is that our data allowed for identification of only around a third of all infected people: those who have had a positive PCR test. The ONS estimates that around 53% of the English population had covid-19 by the end of 2021 [38], equating to 30 m people. Our dataset had 10.5 m unique people with a positive PCR test. Our calculations of risk rates and ratios thus rely on the assumption that SARS-CoV-2 infection rates were the same in both populations.

## Conclusions

Covid-19 carries a disproportionately higher risk of death for people with ID, above the already higher risk of dying from other causes, in comparison to people without ID. Around 2,000 people with ID had a death involving covid-19 in England up to the end of 2021, out of the total population of around 360,000 identified in their GP records as having ID. In comparison with non-ID population, people with ID have had a much higher risk of covid-19 death: 2.1 times in crude terms and 5.6 times in age-standardised terms. This higher risk has been driven mostly by a much higher prevalence of multimorbidity among people with ID. Also, relatively more people with ID happened to be vaccinated with AstraZeneca, which has been somewhat less effective in protecting people with ID than people without ID. However, vaccination in general has been as effective in the ID population as in the non-ID population, which underlines the importance of prioritising this group in vaccine booster programmes.

### Supplementary Information

**Additional file 1: Table A1.** Demographic and health characteristics of people with confirmed SARS-CoV-2 infection (TRE database). **Table A2.** Demographic and health characteristics of people alive on 1 January 2020 who had a primary care record, England (TRE database). **Table A3.** Demographic and health characteristics of adults who died due to covid-19, by ID status (TRE database). **Table A4.** Crude probability of dying due to a cause other than covid-19, by ID status, 2020 (TRE database). **Table A5.** Probability of severe covid-19 in the whole population, by ID status and year (TRE dataset). **Table A6.** Pseudo R-squared and Information Criteria from hierarchical logistic regression predicting the risk of non-covid-19 death in 2020 (TRE dataset). **Table A7.** List of SNOMED CT codes used to identify ID. 

**Additional file 2.** CVD-COVID-UK / COVID-IMPACT Consortium Members.

## Data Availability

The data used in this study are available in NHS England’s Secure Data Environment (SDE) service for England, but as restrictions apply they are not publicly available (https://digital.nhs.uk/services/secure-data-environment-service). The CVD-COVID-UK/COVID-IMPACT programme led by the BHF Data Science Centre (https://bhfdatasciencecentre.org/) received approval to access data in NHS England’s SDE service for England from the Independent Group Advising on the Release of Data (IGARD) (https://digital.nhs.uk/about-nhs-digital/corporate-information-and-documents/independent-group-advising-on-the-release-of-data) via an application made in the Data Access Request Service (DARS) Online system (ref. DARS-NIC-381078-Y9C5K) (https://digital.nhs.uk/services/data-access-request-service-dars/dars-products-and-services). The CVD-COVID-UK/COVID-IMPACT Approvals & Oversight Board (https://bhfdatasciencecentre.org/areas/cvd-covid-uk-covid-impact/) subsequently granted approval to this project to access the data within NHS England’s SDE service for England. The de-identified data used in this study were made available to accredited researchers only. Those wishing to gain access to the data should contact bhfdsc@hdruk.ac.uk in the first instance.

## Abbreviations

AIC: Akaike Information Criterion
BHF: British Heart Foundation
BIC: Bayesian Information Criterion
BSA: Business Services Authority
CHESS: COVID 19 Hospitalisation in England Surveillance System
CI: Confidence Interval
DNACPR: Do Not Attempt Cardiopulmonary Resuscitation
EHR: Electronic Health Record
GDPPR: General Practice Extraction Service Data for Pandemic Planning and Research
GP: General Practice
HDR UK: Health Data Research UK
HES: Hospital Episode Statistics
ICD: International Classification of Diseases
ID: Intellectual Disabilities
IMD: Index of Multiple Deprivation
IQR: Interquartile Range
NHS: National Health Service
ONS: Office for National Statistics
PCR: Polymerase Chain Reaction
PHE: Public Health England
SD: Standard Deviation
SDE: Secure Data Environment
SGSS: Second Generation Surveillance System
SIR: Standardized Incidence Ratio
SMR: Standardized Mortality Ratio
SNOMED CT: Systematized Nomenclature of Medicine Clinical Terms
SQL: Structured Query Language
UK: United Kingdom

## Footnotes

1 This latter estimate was however based on a very small number of deaths. Additionally, the QCOVID study used a very extensive list of ID conditions, which means that the study likely misidentified many people without ID as having ID. This, in turn, inflated the gap in risk between people with Down syndrome and people with ID.

2 We use the term ‘death involving covid-19’ consistently with the ONS definition [12].
