# Supplementary material for: Understanding covid-19 outcomes among people with intellectual disabilities in England
Source: BMC Public Health. 2023 Oct 25;23:2099. doi: 10.1186/s12889-023-16993-x (PMC10601171; doi:10.1186/s12889-023-16993-x)
Supplement: Supplementary file 1 — Additional file 1: Table A1. Demographic and health characteristics of people with confirmed SARS-CoV-2 infection (TRE database). Table A2. Demographic and health characteristics of people alive on 1 January 2020 who had a primary care record, England (TRE database). Table A3. Demographic and health characteristics of adults who died due to covid-19, by ID status (TRE database). Table A4. Crude probability of dying due to a cause other than covid-19, by ID status, 2020 (TRE database). Table A5. Probability of severe covid-19 in the whole population, by ID status and year (TRE dataset). Table A6. Pseudo R-squared and Information Criteria from hierarchical logistic regression predicting the risk of non-covid-19 death in 2020 (TRE dataset). Table A7. List of SNOMED CT codes used to identify ID. [file 12889_2023_16993_MOESM1_ESM.docx]

Additional file 1: Supplementary Tables

*Table A1 Demographic and health characteristics of people with confirmed SARS-CoV-2 infection (TRE database)*

|  | **Adults** | | | **Children (under 18)** | | | **All** | | |
| --- | --- | --- | --- | --- | --- | --- | --- | --- | --- |
|  | **No ID** | **ID** | **p value** | **No ID** | **ID** | **p value** | **No ID** | **ID** | **p value** |
| N in the analysis dataset | 8408930 | 45820 |  | 2247350 | 11055 |  | 10661045 | 56880 |  |
| Age at covid-19 (median [IQR]) | 41.79 [29.95, 54.99] | 40.79 [27.99, 57.81] | <0.001 | 11.97 [8.88, 14.89] | 13.48 [10.66, 15.79] | <0.001 | 35.01 [20.75, 51.05] | 33.77 [20.96, 54.02] | <0.001 |
| Female (%) | 54.0 | 42.2 | <0.001 | 50.4 | 35.9 | <0.001 | 53.3 | 41.0 | <0.001 |
| Ethnicity (%) |  |  | <0.001 |  |  | <0.001 |  |  | <0.001 |
| White | 82.8 | 87.7 |  | 82.3 | 77.7 |  | 82.7 | 85.8 |  |
| Asian | 9.2 | 6.5 |  | 8.7 | 11.9 |  | 9.1 | 7.6 |  |
| Black | 3.7 | 3.1 |  | 2.7 | 4.1 |  | 3.5 | 3.3 |  |
| Mixed | 1.9 | 1.6 |  | 3.9 | 4.2 |  | 2.3 | 2.1 |  |
| Other | 2.4 | 1.0 |  | 2.4 | 2.1 |  | 2.4 | 1.2 |  |
| IMD decile (mean (SD)) | 5.36 (2.85) | 4.59 (2.74) | <0.001 | 5.68 (2.93) | 4.80 (2.87) | <0.001 | 5.43 (2.87) | 4.63 (2.77) | <0.001 |
| Percent vaccinated at least once | 50.8 | 43.3 | <0.001 | 8.8 | 14.7 | <0.001 | 41.9 | 37.8 | <0.001 |
| Vaccine, if any: (%) |  |  | <0.001 |  |  |  |  |  | <0.001 |
| AstraZeneca | 41.4 | 58.7 |  | * | * |  | 39.6 | 54.5 |  |
| Moderna | 6.6 | 2.9 |  | * | * |  | 6.3 | 2.7 |  |
| Pfizer | 51.9 | 38.4 |  | * | * |  | 54.0 | 42.7 |  |
| Percent affected by multimorbidity** | 13.1 | 32.4 | <0.001 | 1.7 | 18.3 | <0.001 | 10.7 | 29.6 | <0.001 |
| Count of Long-Term Conditions (mean (SD))*** | 1.32 (3.18) | 2.60 (4.20) | <0.001 | 0.17 (0.84) | 1.18 (2.64) | <0.001 | 1.08 (2.89) | 2.33 (3.99) | <0.001 |
| Percent affected by polypharmacy**** | 20.9 | 55.6 | <0.001 | 3.4 | 21.8 | <0.001 | 17.3 | 49.0 | <0.001 |
| Count of prescription medications (mean (SD)) | 2.70 (3.84) | 6.28 (5.07) | <0.001 | 0.77 (1.52) | 2.79 (3.49) | <0.001 | 2.29 (3.57) | 5.60 (5.00) | <0.001 |

* Figures supressed due to disclosure rules.

** Multimorbidity: 3+ LTCs (including ID) of which at least one is physical [9].

*** The count did not include ID.

**** 5+ prescription medications

*Table A2 Demographic and health characteristics of people alive on 1 January 2020 who had a primary care record, England (TRE database)*

|  | **Adults** | | | **Children (under 18)** | | | **All** | | |
| --- | --- | --- | --- | --- | --- | --- | --- | --- | --- |
|  | **No ID** | **ID** | **p value** | **No ID** | **ID** | **p value** | **No ID** | **ID** | **p value** |
| N in the analysis dataset | 48,274,905 | 289,085 |  | 12,914,260 | 76,945 |  | 61,189,165 | 366,030 |  |
| Age on 1 Jan 2020 (median [IQR]) | 47.00 [33.00, 63.00] | 39.00 [27.00, 55.00] | <0.001 | 9.00 [4.00, 14.00] | 13.00 [9.00, 16.00] | <0.001 | 39.00 [22.00, 58.00] | 32.00 [20.00, 51.00] | <0.001 |
| Female (%) | 50.3 | 40.6 | <0.001 | 48.9 | 34.6 | <0.001 | 50.0 | 39.4 | <0.001 |
| Ethnicity (%) |  |  | <0.001 |  |  | <0.001 |  |  | <0.001 |
| White | 81.9 | 87.5 |  | 74.2 | 73.2 |  | 80.2 | 84.5 |  |
| Asian | 8.9 | 6.4 |  | 11.9 | 12.9 |  | 9.5 | 7.8 |  |
| Black | 3.9 | 3.3 |  | 5.3 | 6.9 |  | 4.2 | 4.1 |  |
| Mixed | 1.7 | 1.6 |  | 4.6 | 4.2 |  | 2.3 | 2.1 |  |
| Other | 3.7 | 1.1 |  | 4.0 | 2.8 |  | 3.8 | 1.5 |  |
| IMD decile (mean (SD)) | 5.44 (2.84) | 4.54 (2.76) | <0.001 | 5.20 (2.93) | 4.66 (2.85) | <0.001 | 5.39 (2.86) | 4.57 (2.78) | <0.001 |
| Count of Long-Term Conditions (mean (SD))* | 1.00 (2.66) | 1.48 (3.10) | <0.001 | 0.18 (0.80) | 0.89 (2.22) | <0.001 | 0.83 (2.41) | 1.35 (2.94) | <0.001 |
| Percent affected by multimorbidity** | 10.0 | 19.7 | <0.001 | 1.6 | 13.7 | <0.001 | 8.2 | 18.4 | <0.001 |
| Count of prescription medications (mean (SD)) | 2.71 (3.88) | 4.95 (4.70) | <0.001 | 0.62 (1.37) | 2.43 (3.27) | <0.001 | 2.27 (3.60) | 4.42 (4.55) | <0.001 |
| Percent affected by polypharmacy*** | 22.6 | 44.0 | <0.001 | 2.5 | 18.6 | <0.001 | 18.3 | 38.6 | <0.001 |

* The count does not include ID.

** Multimorbidity: 3+ LTCs (including ID) of which at least one is physical.

*** 5+ prescription medications.

*Table A3 Demographic and health characteristics of adults who died due to covid-19, by ID status (TRE database)*

|  | **No ID** | **ID** | **p-value** |
| --- | --- | --- | --- |
| N in the analysis dataset | 160,230 | 2,040 |  |
| Age at covid-19 (median [IQR]) | 82.94 [73.91, 89.17] | 65.57 [56.17, 75.00] | <0.001 |
| Female (%) | 45.1 | 40.9 | <0.001 |
| Ethnicity (%) |  |  | 0.07 |
| White | 88.1 | 89.7 |  |
| Asian | 6.8 | 6.3 |  |
| Black | 3.1 | 2.6 |  |
| Mixed | 0.7 | 0.6 |  |
| Other | 1.4 | 0.7 |  |
| IMD decile (mean (SD)) | 5.12 (2.86) | 4.54 (2.70) | <0.001 |
| Percent vaccinated at least once | 19.2 | 13.8 | <0.001 |
| Vaccine (%): |  |  |  |
| AstraZeneca | * | * |  |
| Moderna | * | * |  |
| Pfizer | * | * |  |
| Count of Long-Term Conditions (mean (SD))** | 10.05 (5.71) | 8.19 (5.42) | <0.001 |
| Percent affected by multimorbidity*** | 86.3 | 85.2 | 0.171 |
| Count of prescription medications (mean (SD)) | 10.80 (4.54) | 10.76 (4.44) | 0.708 |
| Percent affected by polypharmacy**** | 87.8 | 88.9 | 0.147 |

* Figures supressed due to disclosure rules.

** The count does not include ID.

*** Multimorbidity: 3+ LTCs (including ID) of which at least one is physical.

**** 5+ prescription medications.

*Table A4 Crude probability of dying due to a cause other than covid-19, by ID status, 2020 (TRE database)*

|  | ID | No ID |
| --- | --- | --- |
| Population size* | 365,155 | 61,086,510 |
| Number of non-covid-19 deaths | 3,665 | 451,445 |
| Cumulative incidence (%) | 1.00 | 0.74 |
| That is 1 in … | 100 | 136 |
| Crude Risk Ratio | 1.4 | |
| Number of expected non-covid-19 deaths | 1,061 | reference population |
| Non-covid-19 SMR [95% CI] | 3.5 [3.3-3.7] | |

* Number of records in the dataset

*Table A5 Probability of severe covid-19 in the whole population, by ID status and year (TRE dataset)*

|  | 2020 | | 2021 | |
| --- | --- | --- | --- | --- |
|  | ID | No ID | ID | No ID |
| Population size* | 365,155 | 61,086,510 | 365,890 | 61,675,315 |
| Number of cases of severe covid** | 3,835 | 255,740 | 4,085 | 303,560 |
| Cumulative incidence (%) | 1.05 | 0.42 | 1.12 | 0.49 |
| That is 1 in … | 95 | 237 | 88 | 200 |
| Crude Risk Ratio | 2.5 | | 2.3 | |
| Number of expected cases of severe covid-19 | 870 | reference population | 1,227 | reference population |
| Severe covid-19 SIR [95% CI] | 4.4 [4.3-4.5] | | 3.3 [3.2-3.4] | |

* Number in the analysis dataset

** Unique individuals

*Table A6 Pseudo R-squared and Information Criteria from hierarchical logistic regression predicting the risk of* ***non-covid-19 death*** *in 2020 (TRE dataset)*

|  | ID | | | No ID | | |
| --- | --- | --- | --- | --- | --- | --- |
|  | Pseudo R2 | AIC | BIC | Pseudo R2 | AIC | BIC |
| Intercept-only | 0.000 | 38441.3 | -3594224.3 | 0.000 | 5063103.1 | -848032522.7 |
| As above + age | 0.143 | 32945.9 | -3599709.2 | 0.242 | 3836820 | -849258790 |
| As above + sex | 0.143 | 32945.2 | -3599699.3 | 0.244 | 3828191.1 | -849267403.3 |
| As above + ethnicity | 0.143 | 32946.4 | -3599687.5 | 0.244 | 3826851.1 | -849268727.6 |
| As above + IMD decile | 0.143 | 32948 | -3599643.6 | 0.248 | 3807953.6 | -849287562.3 |
| As above + multimorbidity | 0.246 | 28987.7 | -3603593.3 | 0.370 | 3190420.7 | -849905079.5 |
| As above + polypharmacy | 0.273 | 27963.1 | -3604607.3 | 0.398 | 3048954.5 | -850046530 |

*Table A7 List of SNOMED CT codes used to identify ID*

| **SNOMED CT code** | **description** |
| --- | --- |
| 2593002 | Dubowitz's syndrome |
| 5619004 | Bardet-Biedl syndrome |
| 10007009 | Coffin-Siris syndrome |
| 15182000 | Coffin-Lowry syndrome |
| 17122004 | 4p partial monosomy syndrome |
| 17827007 | Cross syndrome |
| 21111006 | Complete trisomy 13 syndrome |
| 21634003 | Borjeson-Forssman-Lehmann syndrome |
| 31216003 | Profound intellectual disability |
| 33982008 | Hyperphosphatasemia with intellectual disability |
| 40354009 | De Lange syndrome |
| 40700009 | Severe intellectual disability |
| 41040004 | Complete trisomy 21 syndrome |
| 51500006 | Complete trisomy 18 syndrome |
| 56604005 | Cohen syndrome |
| 57917004 | Seckel syndrome |
| 59252009 | Cutis laxa-corneal clouding-oligophrenia syndrome |
| 61152003 | Moderate intellectual disability |
| 65327002 | Mucopolysaccharidosis type I-H |
| 66758006 | Acrodysostosis |
| 68618008 | Rett's disorder |
| 70173007 | 5p partial monosomy syndrome |
| 76880004 | Angelman syndrome |
| 79385002 | Lowe syndrome |
| 86765009 | Mild intellectual disability |
| 89392001 | Prader-Willi syndrome |
| 109478007 | Kohlschutter's syndrome |
| 110359009 | Intellectual disability |
| 205615000 | Trisomy 21- meiotic nondisjunction |
| 205616004 | Trisomy 21- mitotic nondisjunction mosaicism |
| 205824006 | Noonan's syndrome |
| 232059000 | Laurence-Moon syndrome |
| 234146006 | Lymphedema lymphangiectasia intellectual disability syndrome |
| 253176002 | Gillespie syndrome |
| 254264002 | Partial trisomy 21 in Down's syndrome |
| 254268004 | Partial trisomy 13 in Patau's syndrome |
| 371045000 | Translocation Down syndrome |
| 401315004 | Smith-Magenis syndrome |
| 403554008 | Oculo-cerebro-cutaneous syndrome (aplasia cutis skin tags eye & brain defects) |
| 412787009 | Intellectual disability congenital heart disease blepharophimosis blepharoptosis and hypoplastic teeth |
| 416075005 | On learning disability register (finding) |
| 422437002 | X-linked intellectual disability with marfanoid habitus |
| 432091002 | Savant syndrome |
| 442511009 | PEHO syndrome |
| 699297004 | Ohdo syndrome, Maat-Kievit-Brunner type |
| 699298009 | Blepharophimosis-mental retardation syndrome Say-Barber-Biesecker-Young-Simpson type |
| 699669001 | Renpenning syndrome |
| 702327009 | Monocarboxylate transporter 8 deficiency |
| 702344008 | Pitt-Hopkins syndrome |
| 702357000 | Chromosome 2q37 deletion syndrome |
| 702412005 | Partington syndrome |
| 702416008 | Snyder-Robinson syndrome |
| 702816000 | Methyl-cytosine phosphate guanine binding protein-2 duplication syndrome |
| 703526007 | Progressive epilepsy-intellectual disability syndrome Finnish type |
| 703535000 | Mowat-Wilson syndrome |
| 715409005 | Trigonocephaly C syndrome |
| 715428003 | Skeletal dysplasia with epilepsy and short stature syndrome |
| 715628009 | Intellectual disability truncal obesity retinal dystrophy and micropenis syndrome |
| 715989002 | Karandikar Maria Kamble syndrome |
| 716024001 | GMS syndrome |
| 716089008 | Craniofacial digital and genital anomalies syndrome |
| 716096005 | Goldblatt Wallis syndrome |
| 716112005 | Kawashima Tsuji syndrome |
| 716191002 | Alopecia and intellectual disability syndrome |
| 716334004 | Intellectual disability and short stature with hand contracture and genital anomaly syndrome |
| 716709002 | FRAXE intellectual disability syndrome |
| 716996008 | L1 syndrome |
| 717157006 | Trisomy 10p |
| 717223008 | X-linked epilepsy with learning disability and behavior disorder syndrome |
| 717763008 | Chudley Lowry Hoar syndrome |
| 717822006 | Goldberg Shprintzen megacolon syndrome |
| 717887003 | Biemond syndrome type 2 |
| 717945001 | BRESEK syndrome |
| 718226002 | Wolf Hirschhorn syndrome |
| 718573009 | Achalasia microcephaly syndrome |
| 718577005 | Atkin Flaitz syndrome |
| 718680001 | Oro-facial digital syndrome type 9 |
| 718681002 | Oro-facial digital syndrome type 11 |
| 718766002 | Spondyloepiphyseal dysplasia, craniosynostosis, cleft palate, cataract and intellectual disability syndrome |
| 718846001 | X-linked intellectual disability Zorick type |
| 718848000 | Fried syndrome |
| 718897009 | X-linked intellectual disability Seemanova type |
| 718900002 | Syndromic X-linked intellectual disability type 11 |
| 718905007 | X-linked intellectual disability Shrimpton type |
| 718908009 | X-linked intellectual disability Siderius type |
| 718909001 | X-linked intellectual disability Stevenson type |
| 718910006 | X-linked intellectual disability Stocco Dos Santos type |
| 718911005 | X-linked intellectual disability Stoll type |
| 718912003 | X-linked intellectual disability Turner type |
| 718914002 | X-linked intellectual disability Van Esch type |
| 719009006 | X-linked intellectual disability Wilson type |
| 719010001 | X-linked intellectual disability Schimke type |
| 719011002 | X-linked intellectual disability Pai type |
| 719012009 | X-linked intellectual disability Miles Carpenter type |
| 719013004 | X-linked intellectual disability Cilliers type |
| 719016007 | X-linked intellectual disability Cantagrel type |
| 719017003 | X-linked intellectual disability Armfield type |
| 719018008 | X-linked intellectual disability Abidi type |
| 719020006 | Pallister W syndrome |
| 719069008 | Shprintzen Goldberg craniosynostosis syndrome |
| 719097002 | BSG syndrome |
| 719136005 | X-linked intellectual disability with cerebellar hypoplasia syndrome |
| 719137001 | X-linked intellectual disability with corpus callosum agenesis and spastic quadriparesis syndrome |
| 719138006 | X-linked intellectual disability with cubitus valgus and dysmorphism syndrome |
| 719139003 | Pettigrew syndrome |
| 719140001 | Prieto Badia Mulas syndrome |
| 719155005 | X-linked intellectual disability and epilepsy with progressive joint contracture and facial dysmorphism syndrome |
| 719157002 | X-linked intellectual disability and hypotonia with facial dysmorphism and aggressive behaviour syndrome |
| 719160009 | Syndromic X-linked intellectual disability type 7 |
| 719162001 | Radioulnar synostosis with microcephaly and scoliosis syndrome |
| 719202006 | Spondyloepiphyseal dysplasia tarda Kohn type |
| 719212004 | Smith Fineman Myers syndrome |
| 719378009 | Microcephalus with brachydactyly and kyphoscoliosis syndrome |
| 719380003 | Microcephalus cardiomyopathy syndrome |
| 719396000 | Microcephalus and intellectual disability with phalangeal and neurological anomaly syndrome |
| 719450007 | Disorder of sex development with intellectual disability syndrome |
| 719599008 | 19q13.11 microdeletion syndrome |
| 719800009 | DOORS syndrome |
| 719808002 | Chromosome Xp11.3 microdeletion syndrome |
| 719810000 | X-linked intellectual disability with seizure and psoriasis syndrome |
| 719811001 | X-linked intellectual disability Cabezas type |
| 719812008 | X-linked intellectual disability with plagiocephaly syndrome |
| 719834005 | Wilson Turner syndrome |
| 719842006 | Congenital hypoplasia of ulna and intellectual disability syndrome |
| 719909009 | Chromosome Xq28 trisomy syndrome |
| 719947004 | Temtamy syndrome |
| 720401009 | Cystic fibrosis with gastritis and megaloblastic anemia syndrome |
| 720468000 | Aniridia and intellectual disability syndrome |
| 720501007 | Arachnodactyly with abnormal ossification and intellectual disability syndrome |
| 720517001 | Ataxia with deafness and intellectual disability syndrome |
| 720523006 | Autosomal recessive limb girdle muscular dystrophy type 2K |
| 720635002 | Cerebro-facio-thoracic dysplasia |
| 720639008 | Coloboma congenital heart disease ichthyosiform dermatosis intellectual disability ear anomaly syndrome |
| 720746006 | Contracture with ectodermal dysplasia and orofacial cleft syndrome |
| 720748007 | Cooper Jabs syndrome |
| 720954000 | Filippi syndrome |
| 720955004 | Fine Lubinsky syndrome |
| 720957007 | Deafness with skeletal dysplasia and lip granuloma syndrome |
| 720958002 | Frank-Ter Haar syndrome |
| 720979002 | Alopecia contracture dwarfism intellectual disability syndrome |
| 720981000 | Alopecia and intellectual disability with hypergonadotropic hypogonadism syndrome |
| 720982007 | Alport syndrome intellectual disability midface hypoplasia elliptocytosis syndrome |
| 721007005 | Hair defect with photosensitivity and intellectual disability syndrome |
| 721008000 | Hall Riggs syndrome |
| 721017000 | Postaxial polydactyly and intellectual disability syndrome |
| 721073008 | Short stature with webbed neck and congenital heart disease syndrome |
| 721087008 | Deafness and intellectual disability Martin Probst type syndrome |
| 721089006 | Dentinogenesis imperfecta short stature hearing loss intellectual disability syndrome |
| 721146009 | Intellectual disability, epilepsy, bulbous nose syndrome |
| 721207002 | Seizure sensorineural deafness ataxia intellectual disability electrolyte imbalance syndrome |
| 721208007 | Ectodermal dysplasia with blindness syndrome |
| 721224008 | Holmes Gang syndrome |
| 721875000 | Juberg Marsidi syndrome |
| 721973006 | Lipodystrophy intellectual disability deafness syndrome |
| 721974000 | Lowry MacLean syndrome |
| 722002002 | Intellectual disability balding patella luxation acromicria syndrome |
| 722003007 | Intellectual disability with cataract and kyphosis syndrome |
| 722031003 | Kapur Toriello syndrome |
| 722033000 | Macrocephaly, short stature, paraplegia syndrome |
| 722035007 | Intellectual disability enteropathy deafness peripheral neuropathy ichthyosis keratoderma syndrome |
| 722037004 | Intellectual disability epileptic seizures hypogonadism and hypogenitalism microcephaly obesity syndrome |
| 722055008 | Oculopalatocerebral syndrome |
| 722065002 | Okamoto syndrome |
| 722110003 | Osteogenesis imperfecta retinopathy seizures intellectual disability syndrome |
| 722111004 | Osteopenia, myopia, hearing loss, intellectual disability, facial dysmorphism syndrome |
| 722209002 | Spastic paraplegia, intellectual disability, palmoplantar hyperkeratosis syndrome |
| 722213009 | Severe X-linked intellectual disability Gustavson type |
| 722281001 | Agammaglobulinemia microcephaly craniosynostosis severe dermatitis syndrome |
| 722282008 | Agenesis of corpus callosum, intellectual disability, coloboma, micrognathia syndrome |
| 722379001 | Congenital cataract with hypertrichosis and intellectual disability syndrome |
| 722380003 | Congenital cataract with intellectual disability and hypogonadotropic hypogonadism syndrome |
| 722454003 | Intellectual disability, craniofacial dysmorphism, hypogonadism, diabetes mellitus syndrome |
| 722455002 | Intellectual disability hypoplastic corpus callosum preauricular tag syndrome |
| 722456001 | Intellectual disability, developmental delay, contracture syndrome |
| 722459008 | Male hypergonadotropic hypogonadism, intellectual disability, skeletal anomaly syndrome |
| 722478008 | Skeletal dysplasia with intellectual disability syndrome |
| 723304001 | Microcephaly, seizure, intellectual disability, heart disease syndrome |
| 723332005 | Isodicentric chromosome 15 syndrome |
| 723333000 | Faciocardiorenal syndrome |
| 723336008 | Fallot complex with intellectual disability and growth delay syndrome |
| 723365002 | Hypotrichosis and intellectual disability syndrome Lopes type |
| 723403008 | Microbrachycephaly, ptosis, cleft lip syndrome |
| 723441001 | Non-progressive cerebellar ataxia with intellectual disability |
| 723501008 | Renier Gabreels Jasper syndrome |
| 723504000 | Ramos Arroyo syndrome |
| 723621000 | Spastic tetraplegia, retinitis pigmentosa, intellectual disability syndrome |
| 723676007 | Severe intellectual disability epilepsy anal anomaly distal phalangeal hypoplasia syndrome |
| 723994004 | Seizures and intellectual disability due to hydroxylysinuria |
| 724001005 | Retinitis pigmentosa intellectual disability deafness hypogenitalism syndrome |
| 724137002 | Macrocephaly obesity mental disability ocular abnormality syndrome |
| 724207001 | Kleefstra syndrome |
| 724228005 | Infantile choroidocerebral calcification syndrome |
| 725140007 | Temple Baraitser syndrome |
| 725163002 | X-linked spasticity intellectual disability epilepsy syndrome |
| 725289009 | 5-amino-4-imidazole carboxamide ribosiduria |
| 725589005 | Bullous dystrophy macular type |
| 725906006 | Intellectual disability Buenos Aires type |
| 725908007 | Neurofaciodigitorenal syndrome |
| 725912001 | X-linked intellectual disability Brooks type |
| 726031001 | Cerebellar ataxia intellectual disability optic atrophy skin abnormalities syndrome |
| 726670008 | Weaver Williams syndrome |
| 726672000 | Short stature, unique facies, enamel hypoplasia, progressive joint stiffness, high-pitched voice syndrome |
| 726709001 | Intellectual disability cataract calcified pinna myopathy syndrome |
| 726727003 | X-linked intellectual disability Hedera type |
| 726732002 | X-linked intellectual disability Nascimento type |
| 732246009 | X-linked intellectual disability, limb spasticity, retinal dystrophy, diabetes insipidus syndrome |
| 732251003 | Cortical blindness, intellectual disability, polydactyly syndrome |
| 732954002 | Osteopenia, intellectual disability, sparse hair syndrome |
| 732961003 | Branchial dysplasia intellectual disability inguinal hernia syndrome |
| 733031004 | Epilepsy microcephaly skeletal dysplasia syndrome |
| 733032006 | Epilepsy telangiectasia syndrome |
| 733049004 | Encephalopathy intracerebral calcification retinal degeneration syndrome |
| 733072002 | Alaninuria microcephaly dwarfism enamel hypoplasia diabetes mellitus syndrome |
| 733086003 | Pseudoprogeria syndrome |
| 733088002 | Preaxial polydactyly, colobomata, intellectual disability syndrome |
| 733090001 | Microcephalus, digital anomaly, intellectual disability syndrome |
| 733097003 | Ichthyosis, intellectual disability, dwarfism, renal impairment syndrome |
| 733110004 | Van den Bosch syndrome |
| 733117001 | Thumb stiffness, brachydactyly, intellectual disability syndrome |
| 733417008 | Facial dysmorphism, macrocephaly, myopia, Dandy-Walker malformation syndrome |
| 733419006 | Metaphyseal dysostosis intellectual disability conductive deafness syndrome |
| 733455003 | Spastic paraplegia, glaucoma, intellectual disability syndrome |
| 733472005 | Microcephalus, glomerulonephritis, marfanoid habitus syndrome |
| 733522005 | Megalocornea with intellectual disability syndrome |
| 734017008 | Ectodermal dysplasia, intellectual disability, central nervous system malformation syndrome |
| 734349003 | Alpha-thalassaemia intellectual disability syndrome linked to chromosome 16 |
| 763136000 | Charcot-Marie-Tooth disease deafness intellectual disability syndrome |
| 763186006 | Grubben, De Cock, Borghgraef syndrome |
| 763320005 | Craniofaciofrontodigital syndrome |
| 763350002 | Intellectual disability obesity brain malformation facial dysmorphism syndrome |
| 763404001 | Ichthyosis alopecia eclabion ectropion intellectual disability syndrome |
| 763615003 | Aortic arch anomaly facial dysmorphism intellectual disability syndrome |
| 763618001 | Wiedemann Steiner syndrome |
| 763626009 | Intellectual disability due to nutritional deficiency |
| 763665007 | Craniodigital syndrome and intellectual disability syndrome |
| 763722004 | Hypotonia, speech impairment, severe cognitive delay syndrome |
| 763741001 | Intellectual disability, alacrima, achalasia syndrome |
| 763742008 | Intellectual disability, polydactyly, uncombable hair syndrome |
| 763743003 | Intellectual disability spasticity ectrodactyly syndrome |
| 763744009 | Intellectual disability brachydactyly Pierre Robin syndrome |
| 763745005 | Intellectual disability Wolff type |
| 763773007 | Macrocephaly and developmental delay syndrome |
| 763795006 | Malan overgrowth syndrome |
| 763797003 | Agenesis of corpus callosum and abnormal genitalia syndrome |
| 763837007 | Oro-facial digital syndrome type 14 |
| 763861000 | Pachygyria intellectual disability epilepsy syndrome |
| 764861005 | Intellectual disability Birk-Barel type |
| 764950001 | Cryptorchidism, arachnodactyly, intellectual disability syndrome |
| 764959000 | Intellectual disability myopathy short stature endocrine defect syndrome |
| 765089003 | Focal epilepsy intellectual disability cerebro-cerebellar malformation syndrome |
| 765170001 | Sodium voltage-gated channel alpha subunit 8-related epilepsy with encephalopathy |
| 765434008 | Human immunodeficiency virus type I enhancer binding protein 2 related intellectual disability |
| 765471005 | X-linked intellectual disability, hypogonadism, ichthyosis, obesity, short stature syndrome |
| 765761009 | Brachydactyly mesomelia intellectual disability heart defect syndrome |
| 766753005 | Nijmegen breakage syndrome-like disorder |
| 766870005 | Epiphyseal dysplasia, hearing loss, dysmorphism syndrome |
| 766871009 | Diencephalic mesencephalic junction dysplasia |
| 768677000 | PPP2R5D-related intellectual disability |
| 770401007 | 10q22.3q23.3 microdeletion syndrome |
| 770411000 | Distal monosomy 19p13.3 |
| 770564004 | Microcephalic primordial dwarfism Alazami type |
| 770566002 | Monosomy 13q14 syndrome |
| 770595006 | Ring chromosome 12 syndrome |
| 770663003 | Tetrasomy 11q24.1 |
| 770678005 | Progressive encephalopathy with edema hypsarrhythmia and optic atrophy-like syndrome |
| 770679002 | Polyneuropathy intellectual disability acromicria premature menopause syndrome |
| 770719004 | 3q27.3 microdeletion syndrome |
| 770750002 | Intellectual disability seizures macrocephaly obesity syndrome |
| 770755007 | Intellectual disability seizures hypotonia ophthalmologic skeletal anomalies syndrome |
| 770901001 | Autosomal recessive intellectual disability motor dysfunction multiple joint contracture syndrome |
| 770907002 | Kagami Ogata syndrome |
| 770908007 | 49XXXYY syndrome |
| 770948004 | Rhizomelic syndrome Urbach type |
| 771072001 | Monosomy 9p |
| 771149000 | Hepatic fibrosis renal cyst intellectual disability syndrome |
| 771262009 | Pseudoleprechaunism syndrome Patterson type |
| 771336003 | Polymicrogyria with optic nerve hypoplasia |
| 771472009 | Developmental and speech delay due to SRY-box 5 deficiency |
| 771512003 | Autism spectrum disorder due to AUTS2 activator of transcription and developmental regulator deficiency |
| 772127009 | White Sutton syndrome |
| 773230003 | Cyclin-dependent kinase-like 5 deficiency |
| 773329005 | CK syndrome |
| 773394007 | Autosomal recessive frontotemporal pachygyria |
| 773400009 | Severe feeding difficulties failure to thrive microcephaly due to ASXL transcriptional regulator 3 deficiency syndrome |
| 773405004 | Intellectual disability with strabismus syndrome |
| 773419004 | Severe intellectual disability short stature behavioral abnormalities facial dysmorphism syndrome |
| 773498006 | Autosomal recessive cerebellar ataxia epilepsy intellectual disability syndrome due to TUD deficiency |
| 773581009 | Intellectual disability craniofacial dysmorphism cryptorchidism syndrome |
| 773665006 | Hypogonadotropic hypogonadism severe microcephaly sensorineural hearing loss dysmorphism syndrome |
| 773670004 | Distal Xq28 microduplication syndrome |
| 773699009 | Pitt Hopkins-like syndrome |
| 773769008 | Ataxia photosensitivity short stature syndrome |
| 773772001 | Rare non-syndromic intellectual disability |
| 774068004 | AHDC1-related intellectual disability obstructive sleep apnea mild dysmorphism syndrome |
| 778011005 | Severe intellectual disability and progressive spastic paraplegia |
| 782676009 | Distal trisomy 18q |
| 782723007 | Severe intellectual disability progressive spastic diplegia syndrome |
| 782736007 | Intellectual disability facial dysmorphism syndrome due to SET domain containing 5 haploinsufficiency |
| 782753000 | Intellectual disability coarse face macrocephaly cerebellar hypotrophy syndrome |
| 782755007 | Primary microcephaly mild intellectual disability young-onset diabetes syndrome |
| 782772000 | Congenital muscular dystrophy with intellectual disability and severe epilepsy |
| 782886007 | Infantile spasms psychomotor retardation progressive brain atrophy basal ganglia disease syndrome |
| 782941005 | Richieri Costa-da Silva syndrome |
| 782945001 | Ophthalmoplegia intellectual disability lingua scrotalis syndrome |
| 783005002 | Severe microbrachycephaly intellectual disability athetoid cerebral palsy syndrome |
| 783174004 | Congenital muscular dystrophy with intellectual disability |
| 783702009 | X-linked intellectual disability due to glutamate ionotropic receptor AMPA type subunit 3 mutations |
| 783703004 | White matter hypoplasia corpus callosum agenesis intellectual disability syndrome |
| 787093004 | Developmental delay facial dysmorphism syndrome due to mediator complex subunit 13 like deficiency |
| 788417006 | Alopecia epilepsy intellectual disability syndrome Moynahan type |
| 816067005 | Diabetes hypogonadism deafness intellectual disability syndrome |
| 838441009 | Mental retardation adducted thumbs shuffling gait aphasia syndrome |
| 840505007 | Down syndrome co-occurrent with leukemoid reaction associated transient neonatal pustulosis |
| 879919001 | Bilateral megalencephaly |
| 879937000 | Alpha-N-acetylgalactosaminidase deficiency type 1 |
| 880065001 | Alpha-N-acetylgalactosaminidase deficiency type 2 |
| 880066000 | Alpha-N-acetylgalactosaminidase deficiency type 3 |
| 880081006 | 12q15 deletion syndrome |
| 890118006 | Mowat-Wilson syndrome due to monosomy 2q22 |
| 890123006 | 3p25.3 deletion syndrome |
| 890130000 | 9q34 deletion syndrome |
| 890221004 | Acrocardiofacial syndrome |
| 890285006 | Bilateral frontal polymicrogyria |
| 890286007 | Bilateral frontoparietal polymicrogyria |
| 890433006 | Cockayne syndrome type 1 |
| 890434000 | Cockayne syndrome type 2 |
| 1003368009 | Molybdenum cofactor deficiency complementation group B |
| 1003373003 | Microcephaly with simplified gyral pattern |
| 1003374009 | Microlissencephaly |
| 1003387003 | Molybdenum cofactor deficiency complementation group C |
| 1003389000 | Mosaic 1q duplication |
| 1003409002 | Maternal 15q11q13 deletion |
| 1010630006 | X-linked complicated corpus callosum dysgenesis |
| 508171000000105 | Severe learning disability |
| 889211000000104 | Specific learning disability |
| 931001000000105 | Significant learning disability |
| 984661000000105 | Mild learning disability |
| 984671000000103 | Moderate learning disability |
| 984681000000101 | Profound learning disability |
| 1089701000000100 | Profound intellectual development disorder without impairment of behaviour |
| 1089701000000100 | Profound intellectual development disorder without impairment of behaviour |
| 1089711000000100 | Profound intellectual development disorder with significant impairment of behaviour |
| 1089711000000110 | Profound intellectual development disorder with significant impairment of behaviour |
| 1089721000000100 | Profound intellectual development disorder with minimal impairment of behaviour |
| 1089721000000100 | Profound intellectual development disorder with minimal impairment of behaviour |
| 1089731000000100 | Profound intellectual development disorder with impairment of behaviour |
| 1089731000000100 | Profound intellectual development disorder with impairment of behaviour |
| 1089741000000100 | Severe intellectual development disorder without significant impairment of behaviour |
| 1089741000000110 | Severe intellectual development disorder without significant impairment of behaviour |
| 1089751000000100 | Severe intellectual development disorder with significant impairment of behaviour |
| 1089751000000110 | Severe intellectual development disorder with significant impairment of behaviour |
| 1089761000000100 | Severe intellectual development disorder with minimal impairment of behaviour |
| 1089761000000110 | Severe intellectual development disorder with minimal impairment of behaviour |
| 1089771000000100 | Severe intellectual development disorder with impairment of behaviour |
| 1089771000000100 | Severe intellectual development disorder with impairment of behaviour |
| 1089781000000100 | Moderate intellectual development disorder without significant impairment of behaviour |
| 1089791000000100 | Moderate intellectual development disorder with significant impairment of behaviour |
| 1089791000000100 | Moderate intellectual development disorder with significant impairment of behaviour |
| 1089811000000100 | Moderate intellectual development disorder with minimal impairment of behaviour |
| 1089811000000100 | Moderate intellectual development disorder with minimal impairment of behaviour |
| 1089821000000100 | Moderate intellectual development disorder with impairment of behaviour |
| 1089821000000110 | Moderate intellectual development disorder with impairment of behaviour |
| 1089831000000100 | Mild intellectual development disorder without significant impairment of behaviour |
| 1089831000000100 | Mild intellectual development disorder without significant impairment of behaviour |
| 1089841000000100 | Mild intellectual development disorder with significant impairment of behaviour |
| 1089841000000100 | Mild intellectual development disorder with significant impairment of behaviour |
| 1089851000000100 | Mild intellectual development disorder with minimal impairment of behaviour |
| 1089851000000100 | Mild intellectual development disorder with minimal impairment of behaviour |
| 1093991000000100 | Mild intellectual development disorder with impairment of behaviour |
| 1093991000000100 | Mild intellectual development disorder with impairment of behaviour |
| 1094001000000100 | Intellectual development disorder without significant impairment of behaviour |
| 1094001000000110 | Intellectual development disorder without significant impairment of behaviour |
| 1094011000000100 | Intellectual development disorder with significant impairment of behaviour |
| 1094011000000110 | Intellectual development disorder with significant impairment of behaviour |
| 1094021000000100 | Intellectual development disorder with minimal impairment of behaviour |
| 1094021000000100 | Intellectual development disorder with minimal impairment of behaviour |
| 1094031000000100 | Intellectual development disorder with impairment of behaviour |
| 1239331000000100 | Significant intellectual disability |
