# Supplementary material for: Understanding covid-19 outcomes among people with intellectual disabilities in England
Source: BMC Public Health. 2023 Oct 25;23:2099. doi: 10.1186/s12889-023-16993-x (PMC10601171; doi:10.1186/s12889-023-16993-x)
Supplement: Supplementary file 2 — Additional file 2. CVD-COVID-UK / COVID-IMPACT Consortium Members. [file 12889_2023_16993_MOESM2_ESM.pdf]

CVD-COVID-UK / COVID-IMPACT Consortium Members (05/09/2023)

British Heart Foundation Data Science Centre
Led by Health Data Research UK

Institution	Member Name
**Addenbrooke's Hospital**	Jon Boyle
**Barts Health NHS Trust**	Alastair Proudfoot
**Barts Health NHS Trust**	Nida Ahmed
**British Heart Foundation**	Dan O'Connell
**British Heart Foundation**	Naomi Herz
**British Heart Foundation**	Nilesh Samani
**British Heart Foundation**	Sonya Babu-Narayan
**British Heart Foundation**	Zainab Karim
**Cancer Research UK**	Jon Shelton
**Cancer Research UK**	Martina Slapkova
**Cancer Research UK**	Rosie Hinchliffe
**Cancer Research UK**	Shane Johnson
**Cardiff University**	Julia Townson
**European Bioinformatics Institute**	Ewan Birney
**European Bioinformatics Institute**	Moritz Gerstung
**Great Ormond Street Hospital**	Katherine Brown
**Guy's and St Thomas' NHS Foundation Trust**	Benjamin Zuckerman
**Guy's and St Thomas' NHS Foundation Trust**	Ernest Wong
**Guy's and St Thomas' NHS Foundation Trust**	Tasanee Braithwaite
**Health Data Research UK/BHF Data Science Centre**	Anna Stevenson
**Health Data Research UK/BHF Data Science Centre**	Annette Jackson
**Health Data Research UK/BHF Data Science Centre**	Cathie Sudlow
**Health Data Research UK/BHF Data Science Centre**	Fionna Chalmers
**Health Data Research UK/BHF Data Science Centre**	James Farrell
**Health Data Research UK/BHF Data Science Centre**	Jemma Austin
**Health Data Research UK/BHF Data Science Centre**	John Nolan
**Health Data Research UK/BHF Data Science Centre**	Kate McAllister
**Health Data Research UK/BHF Data Science Centre**	Lars Murdock
**Health Data Research UK/BHF Data Science Centre**	Lynn Morrice
**Health Data Research UK/BHF Data Science Centre**	Mehrdad Mizani
**Health Data Research UK/BHF Data Science Centre**	Ross Forsyth
**Health Data Research UK/BHF Data Science Centre**	Rouven Priedon
**Health Data Research UK/BHF Data Science Centre**	Samaira Khan

Institution	Member Name
**Health Data Research UK/BHF Data Science Centre**	Steffen Petersen
**Health Data Research UK/BHF Data Science Centre**	Thomas Bolton
**Health Data Research UK/BHF Data Science Centre**	Zach Welshman
**Healthcare Quality Improvement Partnership**	Caroline Rogers
**Healthcare Quality Improvement Partnership**	Mirek Skrypak
**Imperial College London**	Alun Davies
**Imperial College London**	Arunashis Sau
**Imperial College London**	Costas Kallis
**Imperial College London**	Fu Siong Ng
**Imperial College London**	Hannah Whittaker
**Imperial College London**	Ioanna Tzoulaki
**Imperial College London**	Jennifer Quint
**Imperial College London**	Juliette Unwin
**Imperial College London**	Libor Pastika
**Imperial College London**	Petter Brodin
**Imperial College London**	Philip Stone
**Imperial College London**	Safa Salim
**Imperial College London**	Sam Quill
**Imperial College London**	Sarah Cook
**Imperial College London**	Sarah Onida
**Imperial College London (LCP)**	Alistair Marsland
**Imperial College London (LCP)**	Andrew Thompson
**Imperial College London (LCP)**	Sara Holloway
**Imperial College London (LCP)**	Thomas Porter
**INSIGHT**	Alastair Denniston
**Keele University**	Mamas Mamas
**King's College London**	Gayan Perera
**King's College London**	Sarah Martin
**King's College London**	Abdel Douiri
**King's College London**	Adejoke Oluyase
**King's College London**	Ajay Shah
**King's College London**	Alexandru Dregan
**King's College London**	Anna Bone

Institution	Member Name
**King's College London**	Antonio Cannata
**King's College London**	Ben Bray
**King's College London**	Charles Wolfe
**King's College London**	Daniel Bromage
**King's College London**	Dominic Oliver
**King's College London**	Elena Nikiphorou
**King's College London**	Gareth Williams
**King's College London**	Harry Watson
**King's College London**	Irene Higginson
**King's College London**	Javiera Leniz Martelli
**King's College London**	Jayati Das-Munshi
**King's College London**	Joanna Davies
**King's College London**	Johnny Downs
**King's College London**	Katherine Sleeman
**King's College London**	Mevhibe Hocaoglu
**King's College London**	Rachel Cripps
**King's College London**	Richard Killick
**King's College London**	Theresa McDonagh
**King's College London**	Vasa Curcin
**Leeds Teaching Hospitals NHS Trust**	Carin van Doorn
**London School of Economics & Political Science**	Rocco Friebel
**London School of Hygiene & Tropical Medicine**	Arturo de la Cruz
**London School of Hygiene & Tropical Medicine**	Dorothea Nitsch
**London School of Hygiene & Tropical Medicine**	Patrick Bidulka
**London School of Hygiene & Tropical Medicine**	Qiuju Li
**Manchester University NHS Foundation Trust**	Martin Rutter
**NHS England**	Adam Hollings
**NHS England**	Angeliki Antonarou
**NHS England**	Badar Ahmed
**NHS England**	Deborah Lowe
**NHS England**	Efosa Omigie
**NHS England**	Jake Kasan
**NHS England**	Joshua Day

Institutions party to the Data Sharing Agreement with NHS England for access to the data in the Secure Data Environment service for England for CVD-COVID-UK/COVID-IMPACT are: Imperial College London, King's College London, Swansea University, University College London, University of Bristol, University of Cambridge, University of Glasgow, University of Leicester, University of Liverpool, University of Manchester, University of Oxford, University of Sheffield, University of Southampton.

CVD-COVID-UK / COVID-IMPACT Consortium Members (05/09/2023)

British Heart Foundation Data Science Centre
Led by Health Data Research UK

Institution	Member Name
NHS England	Liam Beckingham
NHS England	Russell Healey
NHS England	Sam Hollings
NHS England	Shoaib Ali Ajaib
NHS England	Steve Ball
NHS Lanarkshire	Mark Barber
NHS Scotland	Carole Morris
NICE	Felix Greaves
NICE	Jennifer Beveridge
NICE	Seamus Kent
NICE	Thomas Lawrence
NICE	Vandana Ayyar-Gupta
Office for National Statistics	Ben Humberstone
Office for National Statistics	Camille Harrison
Office for National Statistics	Myer Glickman
Office for National Statistics	Vahé Nafilyan
Queen Mary University of London	Deepti Gurdasani
Queen's University Belfast	Frank Kee
Queen's University Belfast	Raymond Carragher
Royal Brompton and Harefield Hospitals	Paz Tayal
Royal College of Surgeons of England	David Cromwell
Royal Papworth Hospital NHS Foundation Trust	Florian Falter
Royal Papworth Hospital NHS Foundation Trust	Joseph Newman
Royal United Hospitals Bath NHS Foundation Trust	Jennifer Rossdale
St George's University of London	Elijah Behr
St George's University of London	Nuria Sanchez
St George's University of London	Xinkai Wang
Swansea Bay University Health Board	Daniel Harris
Swansea University	Amanda Marchant
Swansea University	Ashley Akbari
Swansea University	Daniel King
Swansea University	David Powell
Swansea University	Elizabeth A Ellins

Institution	Member Name
Swansea University	Fatemeh Torabi
Swansea University	Gareth Davies
Swansea University	Hoda Abbasizanjani
Swansea University	Huw Strafford
Swansea University	Jane Lyons
Swansea University	Julian Halcox
Swansea University	Laura North
Swansea University	Marcos del Pozo Banos
Swansea University	Owen Pickrell
Swansea University	Ronan Lyons
Swansea University	Rowena Griffiths
Swansea University - DATAMIND	Ann John
University College	Robert Aldridge
University College London	Abraham Olvera-Barrios
University College London	Adnan Tufail
University College London	Alasdair Warwick
University College London	Alex Handy
University College London	Alvina Lai
University College London	Ami Banerjee
University College London	Ana Torralbo
University College London	Ana-Catarina Pinho-Gomes
University College London	Andrew Lambarth
University College London	Anthony Khawaja
University College London	Ashkan Dashtban
University College London	Becky White
University College London	Christina Pagel
University College London	Christopher Tomlinson
University College London	David Selby
University College London	Eloise Withnell
University College London	Emma Whitfield
University College London	Eva Keller
University College London	Evaleen Malgapo
University College London	Flavien Hardy

Institution	Member Name
University College London	Floriaan Schmidt
University College London	Freya Allery
University College London	Harry Hemingway
University College London	Honghan Wu
University College London	Jinge Wu
University College London	Johan Thygesen
University College London	Johannes Heyl
University College London	Kate Cheema
University College London	Katie Harron
University College London	Ken Li
University College London	Kerrie Stevenson
University College London	Laura Pasea
University College London	Louise Choo
University College London	Luca Grieco
University College London	Manuel Gomes
University College London	Matt Sydes
University College London	Mehrdad Mizani
University College London	Mel Ramasawmy
University College London	Michalis Katsoulis
University College London	Mohamed Mohamed
University College London	Nushrat Khan
University College London	Paula Lorgelly
University College London	Pedro Machado
University College London	Pia Hardelid
University College London	Qi Huang
University College London	Ravi Shankar
University College London	Riyaz Patel
University College London	Roy Schwartz
University College London	Ruth Gilbert
University College London	Samuel Kim
University College London	**Sonya Crowe**
University College London	Spiros Denaxas
University College London	Tuankasfee Hama

Institutions party to the Data Sharing Agreement with NHS England for access to the data in the Secure Data Environment service for England for CVD-COVID-UK/COVID-IMPACT are: Imperial College London, King's College London, Swansea University, University College London, University of Bristol, University of Cambridge, University of Glasgow, University of Leicester, University of Liverpool, University of Manchester, University of Oxford, University of Sheffield, University of Southampton.

CVD-COVID-UK / COVID-IMPACT Consortium Members (05/09/2023)

British Heart Foundation Data Science Centre
Led by Health Data Research UK

Institution	Member Name
**University College London**	Waty Lilaonitkul
**University College London**	Yat Yi Fan
**University College London**	Yi Mu
**University College London**	Yoryos Lyratzopoulos
**University College London / DATAMIND**	David Osborn
**University Hospital Bristol NHS Foundation Trust**	Serban Stoica
**University Hospital of North Midlands**	Arun Pherwani
**University of Aberdeen**	Mary Joan Macleod
**University of Birmingham**	Sarah Wang
**University of Brighton**	Ravina Barrett
**University of Bristol**	Arun Karthikeyan Suseeladevi
**University of Bristol**	Ben Gibbison
**University of Bristol**	Dann Mitchell
**University of Bristol**	Deborah Lawler
**University of Bristol**	Elsie Horne
**University of Bristol**	Gianni Angelini
**University of Bristol**	Jeremy Chan
**University of Bristol**	John Macleod
**University of Bristol**	Jonathan Sterne
**University of Bristol**	Katharine Looker
**University of Bristol**	Kurt Taylor
**University of Bristol**	Livia Pierotti
**University of Bristol**	Luisa Zuccolo
**University of Bristol**	Martha Elwenspoek
**University of Bristol**	Marwa Al Arab
**University of Bristol**	Massimo Caputo
**University of Bristol**	Mira Hidajat
**University of Bristol**	Neil Davies
**University of Bristol**	Rachel Denholm
**University of Bristol**	Rochelle Knight
**University of Bristol**	Rupert Payne
**University of Bristol**	Shubhra Sinha

Institution	Member Name
**University of Bristol**	Teri-Louise North
**University of Bristol**	Tim Dong
**University of Bristol**	Tom Palmer
**University of Bristol**	Venexia Walker
**University of Cambridge**	Alexia Sampri
**University of Cambridge**	Amir Gavrieli
**University of Cambridge**	Angela Wood
**University of Cambridge**	Carmen Petitjean
**University of Cambridge**	Chriselda Oliver
**University of Cambridge**	David Brind
**University of Cambridge**	Elena Raffetti
**University of Cambridge**	Elias Allara
**University of Cambridge**	Emanuele Di Angelantonio
**University of Cambridge**	Eoin McKinney
**University of Cambridge**	Fabian Falck
**University of Cambridge**	Genevieve Cezard
**University of Cambridge**	Hannah Harrison
**University of Cambridge**	Haoting Zhang
**University of Cambridge**	Isabel Walter
**University of Cambridge**	Jessica Barrett
**University of Cambridge**	John Danesh
**University of Cambridge**	John Ford
**University of Cambridge**	Katie Saunders
**University of Cambridge**	Lisa Pennells
**University of Cambridge**	Mike Inouye
**University of Cambridge**	Robert Fletcher
**University of Cambridge**	Rutendo Mapeta
**University of Cambridge**	Samantha Ip
**University of Cambridge**	Spencer Keene
**University of Cambridge**	**Stephen Kaptoge**
**University of Cambridge**	Tianxiao Wang
**University of Cambridge**	Tom Pape
**University of Cambridge**	Wen Shi

Institution	Member Name
**University of Cambridge**	Xilin Jiang
**University of Cambridge**	Xiyun Jiang
**University of Cambridge**	Yanfan Li
**University of Dundee**	Daniel Morales
**University of Dundee**	David Moreno Martos
**University of Dundee**	Ewan Pearson
**University of Dundee**	Huan Wang
**University of Dundee**	Ify Mordi
**University of Dundee**	Samira Bell
**University of Edinburgh**	Alan Carson
**University of Edinburgh**	Alice Hosking
**University of Edinburgh**	Annemarie Docherty
**University of Edinburgh**	Baljean Dhillon
**University of Edinburgh**	Carlos Sanchez Soriano
**University of Edinburgh**	Caroline Jackson
**University of Edinburgh**	Christian Schnier
**University of Edinburgh**	Claire Tochel
**University of Edinburgh**	Gwenetta Curry
**University of Edinburgh**	Huayu Zhang
**University of Edinburgh**	Michelle Williams
**University of Edinburgh**	Miguel Bernabeu Llinares
**University of Edinburgh**	Niamh McLennan
**University of Edinburgh**	Rebecca Reynolds
**University of Edinburgh**	Richard Chin
**University of Edinburgh**	Steven Kerr
**University of Edinburgh**	Tim Wilkinson
**University of Edinburgh**	Verónica Cabreira
**University of Edinburgh**	William Whiteley
**University of Exeter**	John Dennis
**University of Glasgow**	Angela Henderson
**University of Glasgow**	Clea du Toit
**University of Glasgow**	Colin Berry
**University of Glasgow**	Craig Melville

Institutions party to the Data Sharing Agreement with NHS England for access to the data in the Secure Data Environment service for England for CVD-COVID-UK/COVID-IMPACT are: Imperial College London, King's College London, Swansea University, University College London, University of Bristol, University of Cambridge, University of Glasgow, University of Leicester, University of Liverpool, University of Manchester, University of Oxford, University of Sheffield, University of Southampton.

CVD-COVID-UK / COVID-IMPACT Consortium Members (05/09/2023)

British Heart Foundation Data Science Centre
Led by Health Data Research UK

Institution	Member Name
**University of Glasgow**	Deborah Kinnear
**University of Glasgow**	Dennis Tran
**University of Glasgow**	Filip Sosenko
**University of Glasgow**	Frederick Ho
**University of Glasgow**	Jill Pell
**University of Glasgow**	Naveed Sattar
**University of Glasgow**	Salil Deo
**University of Glasgow**	Sandosh Padmanabhan
**University of Glasgow**	Terry Quinn
**University of Leeds**	Jianhua Wu
**University of Leicester**	Anna Hansell
**University of Leicester**	Anvesha Singh
**University of Leicester**	Atanu Bhattacharjee
**University of Leicester**	Cameron Razieh
**University of Leicester**	Claire Lawson
**University of Leicester**	Clare Gillies
**University of Leicester**	Francesco Zaccardi
**University of Leicester**	Iain Squire
**University of Leicester**	Kamlesh Khunti
**University of Leicester**	Matthew Bown
**University of Leicester**	Sharmin Shabnam
**University of Leicester**	Shirley Sze
**University of Leicester**	Tom Norris
**University of Leicester**	Tom Yates
**University of Leicester**	Umesh Kadam
**University of Leicester**	Yogini Chudasama
**University of Liverpool**	Caroline Dale
**University of Liverpool**	David Hughes
**University of Liverpool**	Maria Sudell
**University of Liverpool**	Mark Green
**University of Liverpool**	Munir Pirmohamed
**University of Liverpool**	Reecha Sofat
**University of Liverpool**	Rohan Takhar

Institution	Member Name
**University of Liverpool**	Ruwanthi Kolamunnage-Dona
**University of Manchester**	Bernard Keavney
**University of Manchester**	Craig Smith
**University of Manchester**	David Jenkins
**University of Manchester**	Evan Kontopantelis
**University of Manchester**	George Tilston
**University of Manchester**	Glen Martin
**University of Manchester**	Joseph Firth
**University of Manchester**	Lamiece Hassan
**University of Manchester**	Lana Bojanić
**University of Manchester**	Matthew Sperrin
**University of Manchester**	Maya Buch
**University of Manchester**	Richard Williams
**University of Manchester**	Ruth Norris
**University of Manchester**	Ruth Watkinson
**University of Manchester**	Sarah Steeg
**University of Manchester**	Simon Frain
**University of Manchester**	Simon Williams
**University of Newcastle**	Camille Carroll
**University of Newcastle**	Dexter Canoy
**University of Nottingham**	Fiona Pearce
**University of Nottingham**	Stephanie Lax
**University of Oxford**	Aashna Uppal
**University of Oxford**	Antonella Delmestri
**University of Oxford**	Ben Goldacre
**University of Oxford**	Ben Lacey
**University of Oxford**	Dani Prieto-Alhambra
**University of Oxford**	Eva Morris
**University of Oxford**	George Nicholson
**University of Oxford**	James Sheppard
**University of Oxford**	Julia Hippisley-Cox
**University of Oxford**	Kazem Rahimi
**University of Oxford**	Lucy Wright

Institution	Member Name
**University of Oxford**	Mark Ashworth
**University of Oxford**	Marta Pineda Moncusi
**University of Oxford**	Milad Nazarzadeh Larzjan
**University of Oxford**	Mohammad Mamouei
**University of Oxford**	Nick Hall
**University of Oxford**	Raph Goldacre
**University of Oxford**	Salma Chaudhry
**University of Oxford**	Sara Khalid
**University of Oxford**	Seb Bacon
**University of Oxford**	Seyed Alireza Hasheminasab
**University of Oxford**	Shishir Rao
**University of Oxford**	Zeinab Bidel Taleshmekaeil
**University of Plymouth**	Marie-Louise Zeissler
**University of Sheffield**	Jen-Yu Amy Chang
**University of Sheffield**	Norman Briffa
**University of Sheffield**	Peter Bath
**University of Sheffield**	Simone Croft
**University of Sheffield**	Suzanne Mason
**University of Sheffield**	Tim Chico
**University of Southampton**	Nazrul Islam
**University of Strathclyde**	Amanj Kurdi
**University of Strathclyde**	Kim Kavanagh
**University of Strathclyde**	Marion Bennie
**University of Strathlcyde**	Tanja Mueller
**University of Warwick**	Harry Wilde
**University of Warwick**	Majel McGranahan
**University of Warwick**	Sebastian Vollmer
**University of York**	Christina van der Feltz-Cornelis
**University of York**	Han-I Wang
**University of York**	Lorna Fraser
**Wellcome Sanger Institute**	Tapiwa Tungamirai
**Wellcome Trust**	Bilal Mateen

Institutions party to the Data Sharing Agreement with NHS England for access to the data in the Secure Data Environment service for England for CVD-COVID-UK/COVID-IMPACT are: Imperial College London, King's College London, Swansea University, University College London, University of Bristol, University of Cambridge, University of Glasgow, University of Leicester, University of Liverpool, University of Manchester, University of Oxford, University of Sheffield, University of Southampton.
